# DDASSQ: An open‐source, multiple peptide sequencing strategy for label free quantification based on an OpenMS pipeline in the KNIME analytics platform

**DOI:** 10.1002/pmic.202000319

**Published:** 2021-08-21

**Authors:** Monika Svecla, Giulia Garrone, Fiorenza Faré, Giacomo Aletti, Giuseppe Danilo Norata, Giangiacomo Beretta

**Affiliations:** ^1^ Department of Excellence of Pharmacological and Biomolecular Sciences University of Milan Milan Italy; ^2^ Unitech OMICs University of Milan Milan Italy; ^3^ Department of Environmental Science and Policy University of Milan Milan Italy; ^4^ Centro Studio Aterosclerosi Bassini Hospital, Cinisello Balsamo Milan Italy

**Keywords:** LFQ, proteomics, search engine, workflow

## Abstract

In this study we investigated the performance of a computational pipeline for protein identification and label free quantification (LFQ) of LC–MS/MS data sets from experimental animal tissue samples, as well as the impact of its specific peptide search combinatorial approach. The full pipeline workflow was composed of peptide search engine adapters based on different identification algorithms, in the frame of the open‐source OpenMS software running within the KNIME analytics platform. Two different in silico tryptic digestion, database‐search assisted approaches (X!Tandem and MS‐GF+), de novo peptide sequencing based on Novor and consensus library search (SpectraST), were tested for the processing of LC‐MS/MS raw data files obtained from proteomic LC‐MS experiments done on proteolytic extracts from mouse ex vivo liver samples. The results from proteomic LFQ were compared to those based on the application of the two software tools MaxQuant and Proteome Discoverer for protein inference and label‐free data analysis in shotgun proteomics. Data are available via ProteomeXchange with identifier PXD025097.

**Abbreviations**
KNIME: Konstanz Information Miner
DDASSQ: *De novo*, Database Assisted, Spectral Search and Quantification
LC: Liquid Chromatography
MS: Mass Spectrometry
HRMS: High Resolution Mass Spectrometry
SILAC: Stable Isotope Labeling by Amino acids in Cell culture (SILAC)
TMT: Tandem Mass Tgs
iTRAQ: isobaric Tags for Relative and Absolute Quantification
OMSSA: Open Mass Spectrometry Search Algorithm
LFQ: Label‐Free Quantification
UPS: Universal Proteomics Standard
PD: Proteome DiscovererTM
MQ: Proteome Discoverer®

## 1 INTRODUCTION

Currently, high resolution mass spectrometry (HRMS) is considered as the most powerful applicable tool for proteomic analysis. The popularity of this technique is due to its high sensitivity and capacity to collect fast and reliable structural information [1].

In this context, shotgun proteomic studies are of great interest to researchers from different scientific fields, especially for those involved in biological disciplines, experimental and clinical medicine and in pharmaceutical science and biopharmaceutics [2].

In these experiments, protein samples are usually digested into peptides by incubation with one protease, typically trypsin [3]. The produced peptides are then analyzed by LC−MS analysis in which a subset of the available precursor ions is sampled by the MS instrument, isolated, and further fragmented in the gas phase to generate fragment ion spectra (MS/MS spectra). The detected peptide sequences and their relative MS data are submitted to computational techniques aimed at determining the identity of their parent proteins (protein inference) as well as their relative or absolute amounts through different computational approaches.

To enhance quantitative MS accuracy, methods based on sophisticated experimental designs such as stable isotope labeling by amino acids in cell culture (SILAC) [4] and isobaric labeling methods including tandem mass tags (TMT), isobaric tags for absolute and relative quantification (iTRAQ) and dimethyl labeling have been introduced [5].

However, due to the additional time needed to carry out sample processing coupled to the elevated costs to perform these procedures, label free quantification (LFQ) strategy remains the prominent option for the analysis of proteomics‐based studies [6].

Different search engines employed for peptide identification, including database search engine assisted Mascot [7], the de novo peptide sequencing softwares Peaks and Novor [8], or freeware/open‐source search engines such as the Andromeda tool included in MQ [9], OMSSA (open mass spectrometry search algorithm) [10], X!Tandem [11], MS‐GF+ [12, 13] and SpectraST [14, 15] have been created, tested and applied in several studies.

In this context, the application of combined multiple engines presents technical and computational challenges, including their heterogeneity in terms of scoring for identification quality control, the propagation of false discoveries, as well as conspicuous informatics challenges related to the different data formats employed by each software. To tackle these hindrances, integration tools like iProphet and Scaffold have been developed [16, 17].

In this context, Vaudel et al. reported SearchGUI, an open‐source graphical user interface that allows to configure and run the freely available search engines OMSSA and X!Tandem [18], and PeptideShaker, a search engine platform for the interpretation of results from multiple search (X!Tandem, MS‐GF+, MS Amanda, OMSSA, MyriMatch, Comet, Tide, Mascot, Andromeda, MetaMorpheus) and de novo (Novor, DirecTag and mzIdentML) engines [19].

Kwon et al. published MSBlender, a statistical method for the integrative analysis, which is based on the conversion of raw search scores from different database‐assisted search engines (InsPecT, Myrimatch, SEQUEST and X!Tandem) into a probability score for every possible PSM, thus accounting for correlation between search scores and estimating false discovery rates, leading to more PSM identifications than any single search engine at the same false discovery rate [20]. The authors showed that increased identifications improved spectral counts for most proteins and allowed the quantification of proteins that would not have been quantified by individual search engines. Of note, they also demonstrated that enhanced quantification contributes to improved sensitivity in protein differential expression analyses [20]. On a similar line, Zhao et al. reported an efficient identification strategy based on the application of multiple peptide search engines, highlighting the similarity between their proteomic results with those of highly accurate RNA‐seq quantifications [21]. Audain et al. reported a bioinformatics solution based on the KNIME/OpenMS platform to compare the performance of protein inference procedures like PIA, ProteinProphet, Fido, ProteinLP, and MSBayesPro using three database search engines Mascot, X!Tandem, and MS‐GF+ [22].

**Statement of significance**

The identification of peptide sequences for protein quantification represents one of the crucial steps in development of shotgun proteomics experiments. Here, we describe the general impact of combining multiple peptide search engines working on different theoretical and applicative principles, on the protein identification and quantitation performance of a pipeline built in an opensource proteomic platform. Therefore, the results are compared with those generated by the two well established proteomic softwares Proteome DiscovererTM and MaxQuant®.

On the same line, taking a conceptual step forward, recently Mohammed and Palmblad developed a theoretical framework and an automated data processing workflow including different peptide identification methods based on a bioinformatic platform known as Taverna [23]. In this study, the scoring results generated by sequence database search (X!Tandem), were compared and combined with those from spectral library search (SpectraST) and de novo sequencing (PepNovo) algorithms, helping the discrimination of corresponding correct and incorrect peptide identifications.

**Highlights**

1. A proteomic workflow system to perform protein quantification after multi‐search engines peptide identification.
2. Protein inference and quantification done after combination of *de novo*, database assisted search and consensus spectral search.
3. Benchmark with two different tools for proteomic studies.

The aim of this study was to evaluate the protein quantification performance of a proteomic pipeline for LFQ analysis based on the concept of combining multiple peptide search engines which work on different theoretical and applicative principles. The sequential combination of the *de nov* *o* peptide sequencing approach (Novor algorithm), of two in silico tryptic digestion assisted database‐searching assisted parsers (X!Tandem and MS‐GF+), and of the consensus library search‐based peptide identification (SpectraST), were tested through their adapter node versions in the open‐source OpenMS software available in the analytics platform KNIME (Konstanz Information Miner) [24, 25]. We will refer to the workflow based on this approach as DDASSQ (De novo, Database Assisted, Spectral Search and Quantification).

Seeking for further insight into the behavior of proteomic workflows in generating LFQ results, we first tested the performance of search engine combinations and evaluated the quantitative result. Then, the corresponding protein LFQ computed on different proteomic datasets was benchmarked and compared with that obtained using two extensively tested and popular software tools,

MaxQuant (MQ) [9] and Proteome Discoverer (PD).

## 2 RESULTS

### 2.1 DDASSQ accuracy: Spike‐in protein datasets

**FIGURE 1:**
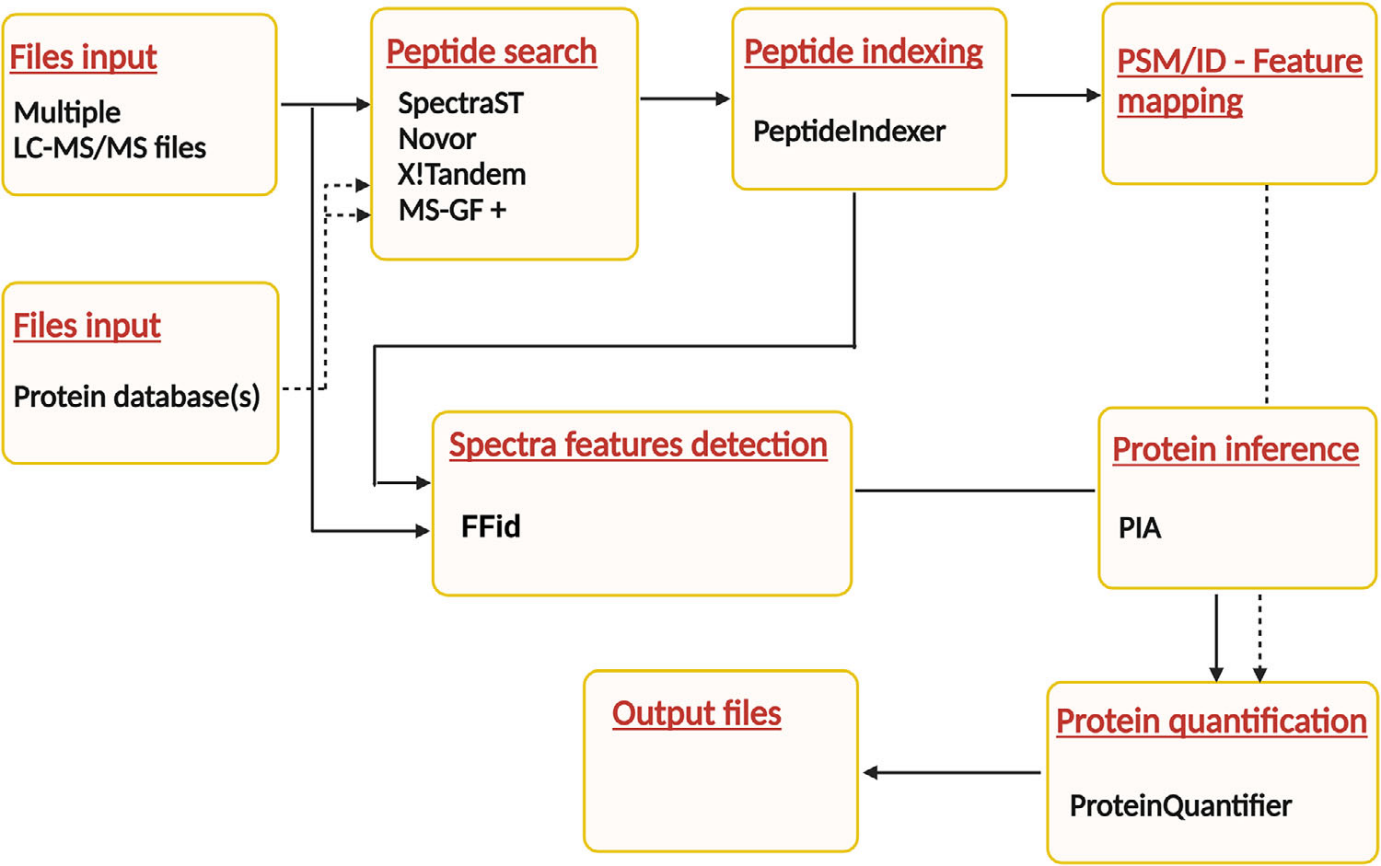
Layout of the tested multiple search engines proteomic OpenMS‐based pipeline

The general structure of the DDASSQ workflow in which LFQ is achieved applying the four peptide search engines X!Tandem, MS‐GF+, Novor and SpectraST, is shown in Figure 1.

**FIGURE 2:**
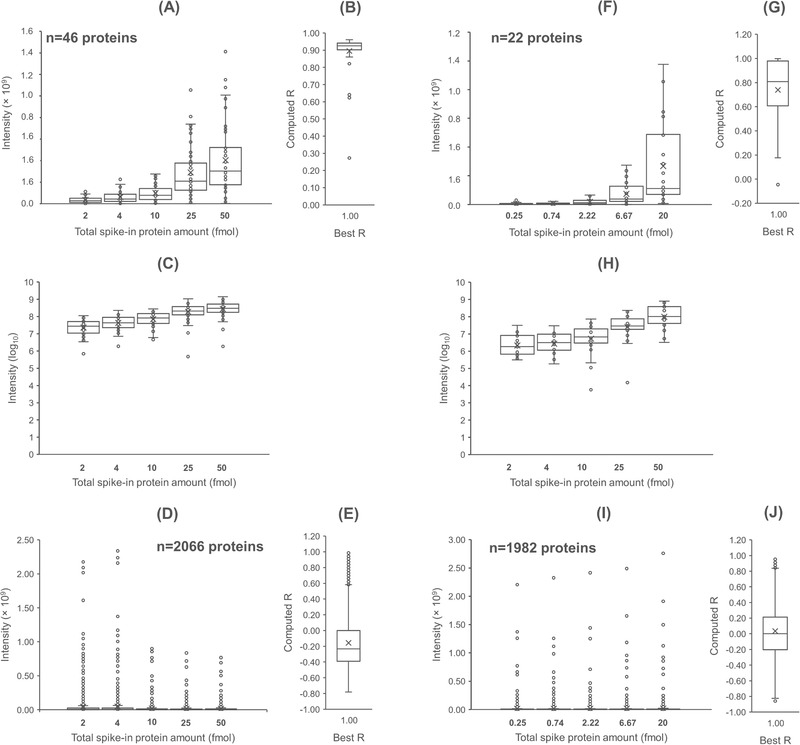
Results of LFQ analysis in DDASSQ workflow. Box‐plot graphs on the left‐hand side: UPS standard proteins (A, F) and *Saccharomyces* *cerevisiae* background proteins (D, I) individual intensities quantified in samples Pursiheimo et al. (ref. [26]) and Tabb et al. (ref. [27], respectively). Graphs C and H: log‐transformed LC‐MS UPS protein intensities. The number of identified spike‐in UPS standard protein and of *S. cerevisiae* background proteins are reported in the corresponding graphs. Side box‐plot graphs (B, E, G, J): pairwise comparison‐based distribution of the correlation coefficients between experimental/theoretical UPS ratios across the tested dilutions for the two sets of proteins, UPS (B, G) and *S. cerevisiae* (E, J) (R‐value computed from experimental data versus best R‐value=1)

The precision and accuracy of the DDASSQ workflow was tested using two datasets published by Pursiheimo et al. (D1) and by Tabb et al. (D2), respectively [26, 27]. These datasets were generated from LC‐MS/MS analysis of samples in which different amounts of UPS standard protein set (equimolar amounts of *n* = 48 *Homo sapiens* proteins) have been added to a background proteome from the yeast *Saccharomyces cerevisiae*. The corresponding protein‐level results are summarized in Figure 2 and the relative data reported in Supplementary material files. In good accordance with the lower concentration range tested in D2 (0.25‐20 fmol/μl) compared to that used in D1 (0.2‐50 fmol/μl), almost all UPS proteins were quantified (*n* = 47/48) in dataset D1, while a lower number of US proteins was identified in dataset D2 (*n* = 25/48).

In both cases, the human protein identified based on the highest number of quantitative peptides was transferrin (gene name: TR; Uniprot accession code: P02787). The identified proteins showed a concentration‐dependent intensity increase (Figure 2A and Figure 2F, and Figure 2C and Figure 2D for log_10_‐tranformed results, respectively), with median correlation coefficient values calculated for their pairwise variation‐ratio (R, Pearson's correlation coefficient) of R_D1_ = 0.9354 and R_D2_ = 0.896 (Figure 2B and Figure 2G, respectively). The proteins with the lowest R‐values were those identified based on a low number of spectra (proteins less susceptible to trypsinization) comparing with those showing high correlation.

Regarding the contribution of yeast background proteome, the corresponding LFQ results trends indicated a progressive decrease of their mean intensity negatively associated with the increasing presence of human UPS proteins. This effect was already detectable in D1, in which a significant difference between the mean intensity of yeast proteins was significantly higher in samples with 2 and 4 fmol/μl of added UPS proteins compared with those with 10–50 fmol/ μl of spike‐in UPS mix (Figure 2D). This effect was more evident in D2, with a non‐significant difference between the lowest tested concentrations only (Figure 2I). Accordingly, the pairwise variation‐ratios corresponding R‐values moved partially toward negative values (Figure 2E and Figure 2L).

The mean percent coefficient of variation (CV%) of the proteins quantified by DDASSQ, MQ and PD workflows in D1 and D2 datasets are reported in Table S1 and Figure S1. When evaluated based on the corresponding individual and mean CV% of the quantified proteins, the performance of the different tools appeared to be dependent on the level of spike‐in protein amount. In the sample with the lowest spike‐in level (0.25 fmol/ μl), the highest number of LFQ intensity CV% was computed based on data generated by PD (*n* = 37; mean CV% = 34.5 %), followed by DDASSQ (*n* = 20; mean CV% = 97.44 %), and by MQ (*n* = 4; mean CV% = 113.4 %). Starting from 10 fmol/ μl concentration, the workflows performance in term of quantified proteins and of their CV% distribution was substantially equivalent (Figure S1).

Quantitative accuracy was evaluated through pairwise comparison‐based analysis of the quantified UPS protein experimental‐to‐theoretical fold increase across the tested spike‐in amounts (Figure S2). MQ results were not included due to a significantly smaller dataset size comparing to those of DDASSQ and MQ.

The analysis evidenced a similar level of accuracy (Figure S2A and Figure S2D), with better performance of PD at lower spike‐in amount range (Dataset D2, Figure 2SA‐C) and higher overall sensitivity of DDASSQ in the higher spike‐in amount range (dataset D1, linear regression slope value: 0.5601, Figure S2E) compared to PD (linear regression slope value: 0.3776 Figure S2F).

### 2.2 Characteristics of in‐house input files

The LC‐MS chromatographic profiles from duplicate analysis of proteolytic peptides obtained from fraction F1 and F2 are reported in Figure S3‐S6. The chromatograms intensities of peaks falling across almost the entire retention time window indicated that the fractionation process led to the recovery of a lower quantitative amounts of peptides in F2 comparing to fraction F1. Under these conditions, it was reasonable to expect differential LFQ values higher in F1 compared to F2.

### 2.3 Proteomic tools performance: General outcomes

The collective results of total number of quantitative proteins and the total number of quantitative peptides identified and selected for LFQ by DDASSQ, PD and MQ are reported in Table 1.

**TABLE 1:** Comparison of the main output characteristics of the proteomic tools run of mouse liver protein extracts

Tool	DDASSQ	PD	MQ
Quantified proteins	3083	1422	1427
Total peptides selected for quantification	21287	9789	10392
Peptide number (mean)	6.90	6.88	7.29
Peptide number (median)	4.00	5.00	5.00
Peptides/protein (max)	106	80	83
LFQ zero values (shared proteins, fraction F2)	1025	186	1123
LFQ zero values (shared proteins, fraction F1)	20	1	38

Statistics are representative of proteins quantified based on at least *n* = 2 unique peptides. OpenMS

Abbreviations: DDASSQ, *de novo*, database assisted, spectral search and quantification; PD, Proteome Discoverer; MQ, MaxQuant.

The results showed that DDASSQ outperformed those of both the tools PD and MQ in terms of almost all parameters, identifying around a double total number of quantifiable proteins (DDASSQ: 3083, PD: 1422 and MQ: 1427) as well as for the number of total identified peptides (DDASSQ: 21287, PD: 9789 and MQ: 10392).

The presence of zero values within a dataset (intensity = 0) is one of the most important LFQ computational problems, especially when statistics of proteins in the low abundance range should be considered. PD was the tool that generated the lowest number of zero values comparing to those generated by DDASSQ and MQ (fraction F2/fraction F1 186/1 vs. 1025/20 and 1123/38, respectively).

MQ ranked first also in terms of mean number of identified quantitative peptide/proteins (7.29 peptides/protein), with 6.90 peptides/protein of DDASSQ and 6.88 peptides/protein of PD (*p* = 0.00007, DDASSQ vs. PD, Student's T‐test). The corresponding median values were identical 4 peptides/protein for DDASSQ and 5 peptides/protein for MQ and PD.

**FIGURE 3:**
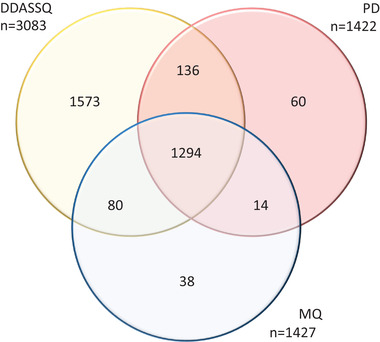
(A) Venn diagram showing the intersection of LFQ protein accessions quantified by DDASSQ, MaxQuant (MQ) and Proteome Discoverer (PD)

The unique and shared identifications are reported as Venn diagram in Figure 3. Out of the *n* = 3083 proteins quantified by at least one software, *n* = 1294 proteins were quantified by all three softwares. The DDASSQ pipeline showed the highest share of proteins quantified by a single tool (1573 accessions), while less than 4% of the total proteins were quantified by PD or MQ only.

**FIGURE 4:**
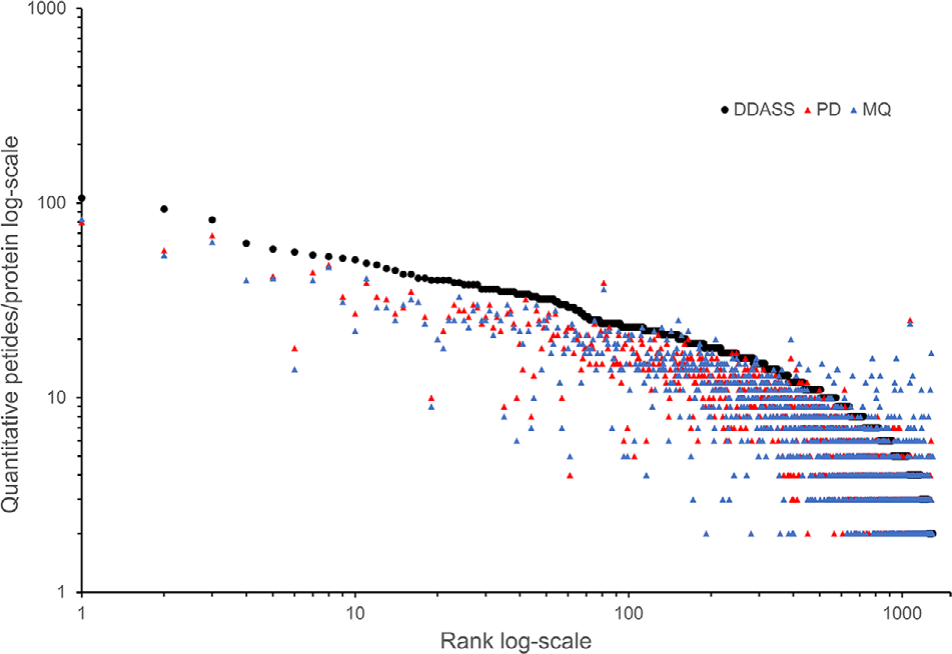
Comparison of DDASSQ, Proteome Discoverer (PD) and MaxQuant (MQ) LFQ quantitative peptides/protein. Protein number *n* = 1294 (shared protein accessions), ranking: DDASSQ.

In Figure 4 are reported the LFQ peptides/protein data for the *n* = 1573 protein accessions included in the LFQ computed by DDASSQ, PD and MQ, ranked by quantitative peptides per protein selected by DDASSQ.

Interestingly, examining the individual results from the graph left‐hand side to the right‐hand side, it emerges that the DDASSQ tool generates a higher number of quantitative peptides per protein compared to PD and MQ. On the other hand, for proteins quantified by DDASSQ based on *n* = 15 peptides and below, PD and MQ often selected a higher number of quantitative peptides.

### 2.4 Impact of search engines combination on protein selection for LFQ

To better understand the contribution of each search engine (i.e., peptide search criteria/approach) to the overall DDASSQ pipeline performance, the workflow was modified by sequential exclusion of the peptide search nodes according to the results layout reported in Table 2. The corresponding individual LFQ and protein inference results are reported in Supplementary material files.

**TABLE 2:** Comparison of the main outputs generated by the OpenMS tool with different peptide search engine combinations from LC‐MS data of trypsinized mouse liver protein extracts

Search engine(s)	Novor	MSGF + Novor	X!Tandem + MSGF+ + Novor	SpectraST + X!Tandem + MSGF+ + Novor
Proteins				
Quantified	52	1607	1742	3083
Mean score	3.27	20.92	20.25	22.33
Peptides				
Total number	153	12111	13616	21287
Mean	2.94	7.54	8.01	6.90
Median	2	5	5	4
Max	14	87	93	106
I_F1_ (×10^11^)	0.08803	2.422	2.52	2.720
I_F2_ (×10^11^)	0.0001316	0.1083	0.124	0.1736

Abbreviation: I_F_, total intensity.

**FIGURE 5:**
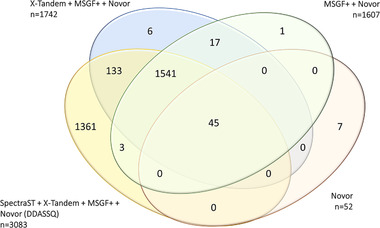
(A) Venn diagram showing the intersections of proteins quantified by the four peptide search engine combinations (see Table 2)

Novor quantified only *n* = 52 protein accessions, corresponding to the 1.70% of the total identification hits. Out of the *n* = 3114 overall unique accessions identified across the protein lists, *n* = 1586 accessions were common to the other tested search engine combinations (31.0 %) (Figure 5).

The introduction of SpectraST in the pipeline was responsible for the 43.7% of the protein identifications reported in the LFQ list.

The X!Tandem and MS‐GF+ contributions were similar, with MS‐GF+ increasing the number of identified peptides per proteins (maximal increment +9 peptides for Carbamoyl‐phosphate synthase [ammonia], mitochondrial; entry Q8C196), simultaneously reducing the total number of identifications due to a lower number of proteins identified based on at least *n* = 2 unique peptides.

The increase in overall number of protein identifications was paralleled by a significant increase in the corresponding total estimated intensities in both fractions F1 and F2, with F1 fraction total intensities higher than those computed for fraction F1 (Table 2).

Taken all together, these results confirm the capacity of the combined peptide search strategies (*de novo* peptide sequencing, database‐assisted search and spectral searching) to yield higher numbers of identified peptides as well as improved identifications, which ultimately should lead also to significant improvements in terms of protein LFQ‐generated quantitative data.

### 2.5 DDASSQ/PD/MQ LFQ correlation results

**FIGURE 6:**
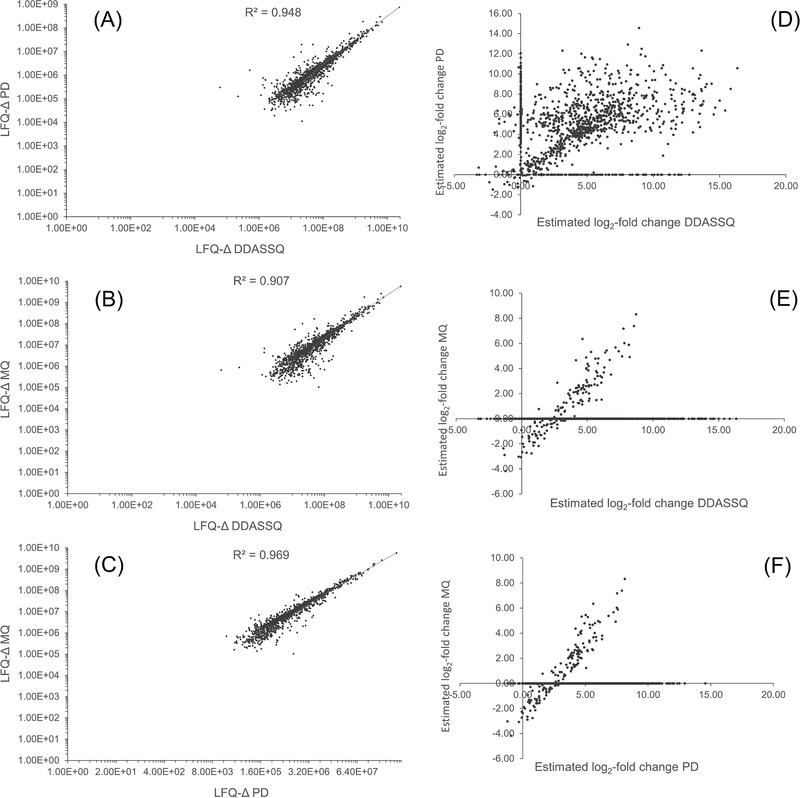
Correlation between DDASSQ, Proteome Discoverer (PD), and MaxQuant (MQ) LFQ results (*n* = 1294 shared quantitative proteins). Results are expressed as log‐transformed intensity variation (LFQ‐Δ, F1 – F2, A‐C) and as log2‐fold change (D‐F)

The concordance of protein LFQ computed by the three proteomic tools DDASSQ, PD and MQ (*n* = 1294 shared proteins) is visualized in Figure 6, both in terms of LFQ intensity variations for each individual protein quantified (LFQ‐Δ, I_F1_‐I_F2_, Figure 6A‐E), and of the corresponding log_2_‐fold variations (Figure 6F‐H).

The scatter plots showed satisfactory correlation between the DDASSQ LFQ‐Δ values of the individual proteins with those computed by PD and MQ, with most data points falling in the first upper‐right quadrant (concordant positive signs), and with similar data point distributions (Figure 6A and Figure 6B, respectively).

The log‐transformed data showed significant correlation between the DDASSQ LFQ‐Δ values with those from PD (R = 0.948, *p* < 0.001, Pearson product‐moment, two‐sided, Figure 6C) and MQ (R = 0.907, *p* < 0.001, Pearson product‐moment, two‐sided, Figure 6D), respectively.

The observed positive variations were in good accordance with what expected based on the adopted sample treatment procedure, in which the original liver protein extract was eluted on a cartridge for specific enrichment of glycoproteins providing a non‐glycosylated protein fraction F1 and a glycoprotein‐enriched fraction F2. Hence, the positive LFQ intensity variations are well explained by the major proportion of non‐glycosylated proteins present in the first washing step, with an average reduction of non‐glycosylated proteins in fraction F2.

LFQ‐Δ values generated by PD and MQ showed excellent correlation (R = 0.969, *p* < 0.001, Pearson product‐moment, two‐sided, Figure 6E).

When expressed as log_2_‐fold variation, the LFQ results showed significant differences in the distribution of those computed by the DDASSQ comparing to those of PD and MQ (Figure 6D‐F). However, data visualization was hindered by the presence of zero values for proteins in F2 fraction in all datasets, with the highest prevalence in the MQ dataset and the lowest in that of PD (see Table 1 for details).

A minor proportion of discordant DDASSQ and PD variations found positive by one tool LFQ and negative by the other one, were observed into the second (*n* = 28 accessions) and fourth (*n* = 9 accessions) graph quadrants (Figure 6F). Most of these proteins ranked in the lower range of quantitative peptides. Hence the apparent discrepancy can be attributed to the random inclusion/exclusion of few peptides implicating discordant intensity variations, an effect similar to that recently reported by Tabb et al. while working on a quantification procedure based on spectral library‐based procedure for the processing of data independent acquisition [27].

Of note, and above all, the best fit linear curves for PD/DDASSQ data showed intercept value close to zero (Figure 6D), while those for MQ/DDASSQ (Figure 6E) and MQ/PD (Figure 6F) showed corresponding negative intercepts, suggesting that in both cases the OpenMS and PD proteomic workflows produce values with *n* = +2 incremental LFQ log_2_ units in respect to those generated by MQ. According to these results, to all the proteins in this range with positive log_2_‐fold value (namely an up‐regulation) found by DDASSQ and PD will correspond a negative value (down‐regulation) assigned by MQ (downregulation). The origin of this apparent systematic error remains to be established.

## 3 DISCUSSION

In the present study, the performance of an LFQ proteomic workflow, based on the combination of three different peptide identification approaches, was evaluated on two different previously reported LC‐MS proteomic datasets as well as on in‐house available dataset obtained from mouse liver protein extracts.

The proposed workflow was built using the OpenMS/KNIME adapters of the peptide search engines Novor, X!Tandem, MS‐GF+ and SpectraST, all working through their specific nodes developed in the KNIME platform [24, 25].

Recent studies reported the impact of the combination of some database‐assisted peptide search tools, working independently within online or in installation‐based computational platforms on the identification of different peptide sequences. This approach increased peptide identification and protein amino acid sequence coverage, thus providing a relatively simple but efficient way to maximize the utilization of mass spectra through the combination of such combined peptide search engines [18, 19, 20, 21, 22, 23].

From the quantitative point of view, the results reported support the concept that the improvement obtained by the application of multiple search engines strategy translates in a more accurate protein quantification, taking advantage of the higher number of proteins identified, with a performance similar to that of highly accurate RNA‐seq approaches [21].

Based on these aspects, we aimed to test a composite proteomic workflow according to the hypothesis that its overall identification and quantification capacity at the proteome level can be improved by the combination of multiple peptide search tools based on radically different theoretical and informatic backgrounds, in line with the hypothesis proposed by Mohammed and Palmblad [23].

One of the goals was to design a flexible, user‐friendly computational system allowing the management of several parameters involved in proteomic pipeline nodes without requiring deep knowledge of their underlying informatic grounds. From this point of view, the OpenMS tools built in the KNIME platform seemed to be an ideal starting point.

Therefore, among those available in the OpenMS/KNIME platform, we first selected the adapter of Novor, one of the commercial software packages working on an algorithm which allows *de novo* peptide sequencing: that is, peptide sequencing is deduced directly from MS/MS data without requiring reference sequence database(s) [8].

The *de novo* peptide sequencing was combined with two database‐assisted search algorithms (X!Tandem and MS‐GF+). X!Tandem, as reported in its original version by Craig and Beavis [11] searches peptide structures starting from tandem MS/MS spectra with the aid of in silico tryptic digestion of target protein sequences. Beside X!Tandem, the more recent sequence database‐assisted sequencing search engine MS‐GF+ tool was included in the combination [12, 13]. One significant advantage of this search engine relies in its insensitiveness to the individual experimental set‐up (low/high resolution, fragmentation mode), improving the identification performance compared to that of other informatic tools designed for specific instrumental solutions [13].

The fourth approach selected was that of SpectraST, a search tool developed by Lam and colleagues that employs spectral searching of the experimental data against a library of experimental annotated MS/MS spectra [14]. According to the authors, this procedure vastly outperforms the identification capacity of the sequence search engine SEQUEST, both in terms of computational speed and of ability to discriminate good and bad hits [14, 15].

The combined identifications were used in the workflow for spectral features definition using the FFId algorithm reported by Weisser and colleagues [29] and subsequent protein inference for protein groups determination, and in parallel for PSMs extraction using the algorithm PIA described by Uszkoreit and colleagues [30, 31]. The choice of FFid over other spectral feature identifiers was done based on its higher capacity in producing quantifiable proteins and its higher speed compared to other analogue tools in OpenMS environment, such as FeatureFinderCentroid. Protein quantification was then achieved through the ProteinQuantifier node, with an approach similar to that described by Silva et al.[32].

In all considered cases, computational descriptors (e.g., total number of identified peptides and proteins) of the LFQ were generally comparable or superior to those obtained using two common proteomic tools such as PD and MQ and X!Tandem, MS‐GF+ in combination with Novor.

The obtained results agreed with previous findings on the determination of liver proteome of mouse strains with different genetic background [33].

On the other hand, the quantification accuracy evaluated through individual and mean quantified protein CV% suggested a substantial equivalence of DDASSQ, PD, and MQ results when applied to datasets with target proteins in the higher concentration range, leading to similar CV% value intervals and quantified protein numbers. By contrast, in the lower concentration range, PD seemed to generate the higher level of sensitivity and accuracy based on the highest observed number of quantified proteins associated to the lowest mean CV% values.

Indeed, these variations may originate from the different peptide identification procedures adopted by the different proteomic tools, as well as from their different criteria of peak area extraction and subsequent data treatment involved in the quantification algorithms. For this reason, further research for the better understanding of the relative contribution of these two factors, alone or in combination, to the increase of uncertainty in the quantification of less responsive proteins, is warranted.

The significant increase of identified peptides/proteins observed in the present study agreed with that reported by Shteynberg et al., that reported an increase in the number of correctly identified peptides when SpectraST results were included in the iProphet combination of those from seven different database‐assisted search engine algorithms [34]. Taken altogether, these results confirm the high sensitivity of SpectraST peptide identification in case of datasets for which high‐quality spectral libraries are available [14, 34].

Recent and excellent studies on the effect of combinatorial approaches involving different types of search algorithms have been reported. However, to the best of our knowledge no study evaluating the impact of spectral searching inclusion on proteomic LFQ, is reported in the literature.

For this reason, to better define the role of spectral searching in the performance of DDASSQ approach, future work will focus on expanding the application of this tool to a wider set of raw data with particular emphasis on the different tissue and cell type, the sample processing procedure and data file dimension.

## 4 CONCLUSIONS

In recent years, admirable advances in LC‐HRMS techniques, together with the availability of more powerful informatic hardware, increased the demand for bioinformatic tools for the efficient management of MS‐based peptide sequencing, protein inference and LFQ methods which is also impacted by the massive and increasing size of the raw data files associated to the results of shotgun proteomic experiments.

In the present study, a combination of peptide identification engines has been evaluated through the flexible OpenMS adapters built in the KNIME environment (an open‐source platform in continuous evolution and optimization).

The results confirm the additional benefit of combining peptide search engines in terms of identification number and robustness, implying that the application of tools based on different theoretical and applicative rules, such in the case of our DDASSQ, results in a further boost of the identification capacity.

Nevertheless, the results of the present study highlight the need for further work and investigations in this specific area of proteomics. In addition to the possible implementation of the available peptide search proteomic nodes in terms of adherence to the MS acquisition experimental conditions (e.g., acquisition mode and fragmentation system), increasing the availability of spectral consensus databases currently limited to a small number of species, will allow more feasible the application of algorithms such as that used by SpectraST; this calls for further extensive work of spectra collection and compilation.

## 5 EXPERIMENTAL SECTION

### 5.1 Chemicals and reagents

All chemicals and supplies used for LC‐MS sample processing were of MS‐grade purity. Water and acetonitrile (ACN) both containing 0.1% formic acid or aqueous trifluoroacetic acid (TFA), were purchased from Carlo Erba Reagents (Carlo Erba Reagents S.r.l., Milan, Italy). Acetone, proteomic grade trypsin (code T7575), dithiothreitol (DTT), iodoacetamide (IAA), ammonium bicarbonate (ambic), urea 8.0 M solution and 0.1 M Tris‐HCl buffer were all purchased from (Sigma‐Aldrich, Milan, Italy). ZipTips were from Thermo Scientific (product code 87784, Thermo Scientific, Rodano, Italy).

### 5.2 Data sets

Computations were run on dataset representing the LC‐MS analysis of tryptic digests of protein extracted from mice liver fed a cholesterol enriched diet [35] and processed as described in the next paragraphs.

### 5.3 Animals

Wild type (WT), male mice on C57BL/6J background were purchased from Charles River (Italy) and The Jackson Laboratory (USA). Old mice (6‐8 weeks old) were fed a high cholesterol diet (western type diet ‐ WTD, E15775‐34 ssniff Spezialdiäten GmbH, DE) for 8 weeks [35]. Mice (*n* = 4 per group) were housed in cages kept in a temperature‐controlled environment (20 ± 2°C, 50 ± 5% relative humidity) with a 12‐h light/dark cycle and free access to food and water [36]. Mice were sacrificed at 20 weeks, after isoflurane (2%) inhalation and cervical dislocation. Livers were explanted and weighted. All animal procedures performed, were done in agreement to the guidelines from 2010/63/EU directive of the European Parliament on the protection of animals used for scientific purposes and were approved by the local Ethical Committee (Progetto di Ricerca 2012/02, Autorizzazione Ministeriale 811/2017).

### 5.4 Sample preparation

Liver segments from WT mice (*n* = 2) were cleaned with sterile ice‐cold PBS 1 × and approximately 10 mg were lysed in the presence of binding buffer, protease inhibitor cocktail and detergent solution at room temperature using Qproteome Total Glycoprotein Kit (Qiagen S.r.l., Milan, Italy). Samples were homogenized with TissueRuptor for 30s at the lowest speed, followed by incubation of the lysate for 15 min at 4°C. Subsequently, samples were centrifuged at 10,000 × *g* for 20 min at 4°C and the supernatant was collected. The lysate was transferred to a spin column and processed according to the manufacturer instructions (http://wolfson.huji.ac.il/purification/PDF/Lectins/QIAGEN_GlycoproteinFractionHandbook.pdf) to obtain non glycosylated protein in flow through solution (F1) and the enriched glycosylated protein fraction (F2). The protein content was measured as described [37]. Cold acetone was added to samples in proportion 4:1 (v/v) and incubated for 15 min in ice. Samples were then centrifuged (12,000 × *g*, 10 min at 4°C), the supernatants were discarded, and the protein pellets resuspended in urea 8.0 M solution and 0.1 M Tris‐HCl buffer (pH 8.5). An additional Lowry protein assay was performed to confirm the protein content after precipitation. Samples were then dried completely using a vacuum concentrator (45°C, 45 min) and resuspended in 5.0 mM DTT in 50 mM ambic buffer (pH 8.5, 30 min at 50°C under mechanical agitation). Samples were then cooled down to RT and alkylation performed by addition of 150 mM IAA in ambic buffer 50 mM (15 mM final concentration) and incubated in the dark for 20 min at RT [38]. Trypsin was added at an enzyme‐to‐protein ratio of 1:20 and the digestion was performed overnight at 37°C, under agitation under mechanical agitation (600 rpm). Medium pH was in the range 8‐8.5 pH units. The digestion was stopped by sample acidification with 50% TFA (final concentration: 1%). Final protein concentration was 0.33 μg/μl. The proteolytic peptide mixtures were purified by C18 pipette tips (ZipTip) and analyzed in duplicate by nano‐liquid chromatography MS/MS (nLC‐MS/MS).

### 5.5 LC‐MS/MS analysis

Samples were analyzed at Unitech OMICs (University of Milano, Italy), using a Dionex Ultimate 3000 nano‐LC system (Sunnyvale CA, USA) connected to an Orbitrap Fusion Tribrid Mass Spectrometer (Thermo Scientific, Bremen, Germany) and equipped with a nano‐ESI ion source. Peptide mixtures were pre‐concentrated onto an Acclaim PepMap C18, 5 μm, 100 Å, 100 μm ID x 2 cm (Thermo Scientific) and separated at 35°C on an EASY‐Spray PepMap RSLC C18 column (3 μm, 100 Å, 75 μm ID × 15 cm; Thermo Scientific). Elutions were run in gradient mode from 96% buffer A (0.1% formic acid in water) to 40% buffer B (0.1% formic acid in water/acetonitrile (20/80 v/v). Total gradient: 110 min. Flow rate: 300 nl/min. Total run time: 144 min. MS acquisition was done in in positive ion mode over an m/z range of 375–1500 Da at 120,000 resolution in the data dependent mode, cycle time 3 s between master scans. MS/MS spectra were collected in centroid mode. Higher collision decomposition (HCD) energy: 35 eV.

### 5.6 DDASSQ workflow

Prior to data analysis, each LC‐MS raw file was converted from raw to mzML format in centroid mode using the MSconvert tool of the software ProteoWizard (version 3.0.1957) [39]. The mzML files were analyzed using a pipeline adapted from Weisser et al. [40], built using OpenMS [25] (version 2.5.0) operating within the open‐source software platform KNIME (version 4.1.3, available at https://www.knime.com/). Spectral search with SpectraST was run using the NIST_mouse_IT_2012‐04‐21_7AA.splib, NIST_human_IT_2012‐05‐30_7AA.splib and NIST_yeast_IT_2012‐04‐06_7AA.splib files were appropriate and downloaded at the URL http://www.peptideatlas.org/speclib/. Human and yeast spectral libraries were concatenated in a single consensus library using the specific command lines in available in SpectraST (http://tools.proteomecenter.org/wiki/index.php?title = Software:SpectraST). Peptide identification was done using a multiple search engine pipeline combining X!Tandem algorithm [11], (XTandemAdapter node), MS‐GF+ [12, 13], Novor (for peptide *de novo* identification) [8] and the MS/MS spectral search tool SpectraST (SpectraSTSearchAdapter node) [14, 15]. X!Tandem, MS‐GF+ search and peptide indexing were done against a mouse FASTA Swiss‐Prot reviewed protein sequence database (uniprot‐filtered‐organism_Mus.musculus‐(Mouse)‐[10090] (*n* = 17046 entries), downloaded at www.uniprot.org (October 2020), including in the protein database a list of common contaminant proteins (*n* = 179, https://github.com/pwilmart/fasta_utilities/blob/master/Thermo_contams.fasta). To this database, for subsequent FDR computation, a decoy reverse sequence database was appended by application of the DecoySequence OpenMS node. For all search engines except SpectraST, cysteine carbamidomethylation was set as fixed modification and methionine oxidation was set as variable modification. Fragment mass tolerance was set at 0.02 Da and precursor mass tolerance at 5.0 ppm. Peptide sequences were indexed through the OpenMS Peptide Indexer node, setting leucine/isoleucine equivalence. Protein inference was carried out using the Protein Inference Algorithms (PIA, version 1.3.11) node [31, 32]. The parameters settings of all individual nodes are reported in Appendix S1. Protein abundance estimates were calculated with prior generation of spectral features by the node FeatureFinderIdentification (FFid) [29] followed by PIA‐assisted FDR estimation and filtering at PSM level (PSM combined FDR score > 0.01, equivalent to FDR < 1%) with subsequent further filtering at peptide and protein group level through IDfiter node options (FDR < 1%), their ID mapping and combination with peptide IDs, their subsequent grouping and normalization (e.g., FeatureLinkerUnlabeledQT and ConsensusmapNormalizer nodes) [38]. Proteins and peptides LFQ was then computed with the OpenMS ProteinQuantifier node based on intensities of all quantitative proteotypic peptide intensities (quantitative peptide number equal/greater than *n* = 2) [32].

The relative output files, read as tables of the CSVreader node output, exported in Microsoft Office Excel 2016 for further formatting and statistical elaboration. Detailed pipeline parameters are shown in Appendix S1, and the full DDASSQ pipeline is available for downlod at the Github.com website at the URL: https://github.com/giangiacomoberetta1/GBeretta.

The mass spectrometry proteomics data have been deposited to the ProteomeXchange Consortium via the PRIDE [41] partner repository with the dataset identifier PXD025097.

### 5.7 Benchmark proteomic softwares

Proteomic data analysis was done using the softwares Proteome Discoverer (PD, version 2.2, Thermo Fisher Scientific, Waltam, MA, USA) and MaxQuant (MQ, version 1.6.7.0) [9]. The PD corresponding data processing workflow is described in the Appendix S2. Both PD and MQ analyses were run using a precursor mass tolerance of 5 ppm and fragment mass tolerance of 0.02 Da, carbamidomethyl as fixed modification and methionine oxidation as variable modification, with the same sequence database used for X!Tandem and MS‐GF+ in the OpenMS workflow. Decoying was done in reverse sequence mode. Trypsin was selected for in silico protein digestion, *n* = 2 maximum number of missed cleavages, peptide length for unspecific search between *n* = 8 and *n* = 25 amino acids, and MQ LFQ and stabilize large LFQ options on. MQ iBAQ was not activated.

### 5.8 Datasets from PRIDE repository

The DDASQ workflow identification and quantification performance was tested using the datasets from two different studies. The first one was published by Pursiheimo et al. and consisted of 2, 4, 10, 25 and 50 fmol/μl UPS spiked to 100 ng of yeast *S*. *cerevisiae* background proteins analyzed by HPLC‐MS/MS using an LTQ Orbitrap Velos MS (*n* = 3 technical replicates of each concentration) [26]. An LTQ Orbitrap Velos MS was used to analyze three technical replicates of each concentration. The corresponding raw data are available from the PRIDE archive with the identifier PXD002099 (http://www.ebi.ac.uk/pride/archive/projects/PXD002099). The second dataset, published by Tabb et al., included triplicate LC‐MS analyses of 0.25, 0.74 2.22, 6.67 and 20 fmol/ UPS μl added to 60 ng of *S*. *cerevisiae* background proteins [27]. Raw data are available for download at the URL https://cptac‐data‐portal.georgetown.edu/cptac/study/showDetails/10424 (sample set Orbi2).

### 5.9 Statistics

For simplicity, the final quantitative LFQ results from duplicate analyses were averaged. Missing intensity values in PD output were converted to zero values. Statistical analysis and graphical data presentation were done using the software Graph Pad‐Prism8 (GraphPad Software, San Diego, CA, USA). Venn diagrams were built with aid of the dedicated tool published online by the Bioinformatic and Evolutionary Genomics group (VIB, Ghent University) available at the URL bioinformatics.psb.ugent.be/webtools/Venn/.

## CONFLICT OF INTEREST

The authors declare no conflict of interest.

## AUTHOR CONTRIBUTIONS

Monika Svecla: experimental procedures, samples processing, manuscript writing—original draft, review and editing, conceptualization. Giulia Garrone: proteomics analysis, software, manuscript review and editing. Methodology. Fiorenza Farè: proteomic analysis, software, manuscript review and editing. Giacomo Aletti: software, formal analysis, supervision. Giuseppe Danilo Norata: review and editing, supervision, conceptualization. Giangiacomo Beretta: conceptualization, software, data curation, formal analysis, supervision, manuscript writing, review and editing.

## Supporting information

Supporting Information

Supporting information

## Data Availability

The data that support the findings of this study are openly available in PRIDE repository with reference number PXD025097, at http://www.ebi.ac.uk/pride/archive/projects/PXD002099 with reference number PXD002099, and at the URL https://cptac‐data‐portal.georgetown.edu/cptac/study/showDetails/10424 (sample set Orbi2).
