# Supplementary material for: DDASSQ: An open‐source, multiple peptide sequencing strategy for label free quantification based on an OpenMS pipeline in the KNIME analytics platform
Source: Proteomics. 2021 Aug 21;21(16):2000319. doi: 10.1002/pmic.202000319 (PMC8459258; doi:10.1002/pmic.202000319)

**Supplementary material**

**DDASSQ: an open-source, multiple peptide sequencing strategy for label free quantification based on an OpenMS pipeline in the KNIME analytics platform**

Monika Svecla^1^, Giulia Garrone^2^, Fiorenza Faré^2^, Giacomo Aletti^3^, Giuseppe Danilo Norata^1,4^, Giangiacomo Beretta^3^

^1^Department of Excellence of Pharmacological and Biomolecular Sciences, University of Milan, Milan, Italy.

^2^Unitech OMICs, University of Milan, Milan, Italy.

^3^Department of Environmental Science and Policy, University of Milan, Milan, Italy.

^4^Centro Studio Aterosclerosi, Bassini Hospital, Cinisello Balsamo, Milan, Italy.

Corresponding author:

Giangiacomo Beretta, Via Mangiagalli 25, 20133 Milan, Italy

Email: [giangiacomo.beretta@unimi.it](mailto:giangiacomo.beretta@unimi.it)

**Table S1**. Comparison of the mean percent coefficient of variation (CV%) of LFQ intensities determined from results computed by DDASSQ, Proteome Discoverer^TM^ and MaxQuant®. Raw LC-MS data were taken from two different studies (D2, Tabb et al. (2010) - CPTAC study ref. [28], and D1, Pursiheimo et al. (2015) – PRIDE repository code PDX002099, ref. [27]). The number of quantified proteins is indicated in parentheses.

| D2 | Spike-in concentration (fmol-µL) | | | | |
| --- | --- | --- | --- | --- | --- |
|  | 0.25 | 0.74 | 2.22 | 6.67 | 20 |
| Mean CV% |  |  |  |  |  |
| DDASSQ (22) | 97.4 (20) | 91.2 (22) | 63.6 (22) | 31.3 (21) | 27.4 (22) |
| Proteome Discoverer^TM^ | 34.5 (37) | 25.5 (39) | 19.5 (43) | 12.3 (44) | 16.3 (44) |
| MaxQuant® | 113.4 (4) | 144.6 (13) | 89.5 (29) | 61.0 (39) | 28.6 (42) |
|  |  |  |  |  |  |
| D1 | Spike-in concentration (fmol-µL) | | | | |
| PXD002099 | 2 | 4 | 10 | 25 | 50 |
| Mean CV% |  |  |  |  |  |
| DDASSQ | 28.49 (47) | 72.31 (46) | 33.74 (46) | 21.32 (46) | 32.37 (46) |
| Proteome Discoverer^TM^ | 14.9 (46) | 37.0 (46) | 26.3(46) | 19.8 (47) | 27.6 (47) |
| MaxQuant® | 40.20 (27) | 104.27 (34) | 35.17 (44) | 21.58 (45) | 18.56 (45) |

**Fig. S1**. Graphical representation of individual mean percent coefficient of variation (CV%) vs. spike-in amount of the protein entries quantified by DDASSQ (n=22, reddish line), PD (n=37, orange line), and MQ (n=4, blue line) in datasets D1 and D2. Values are mean ± SD (n=3).


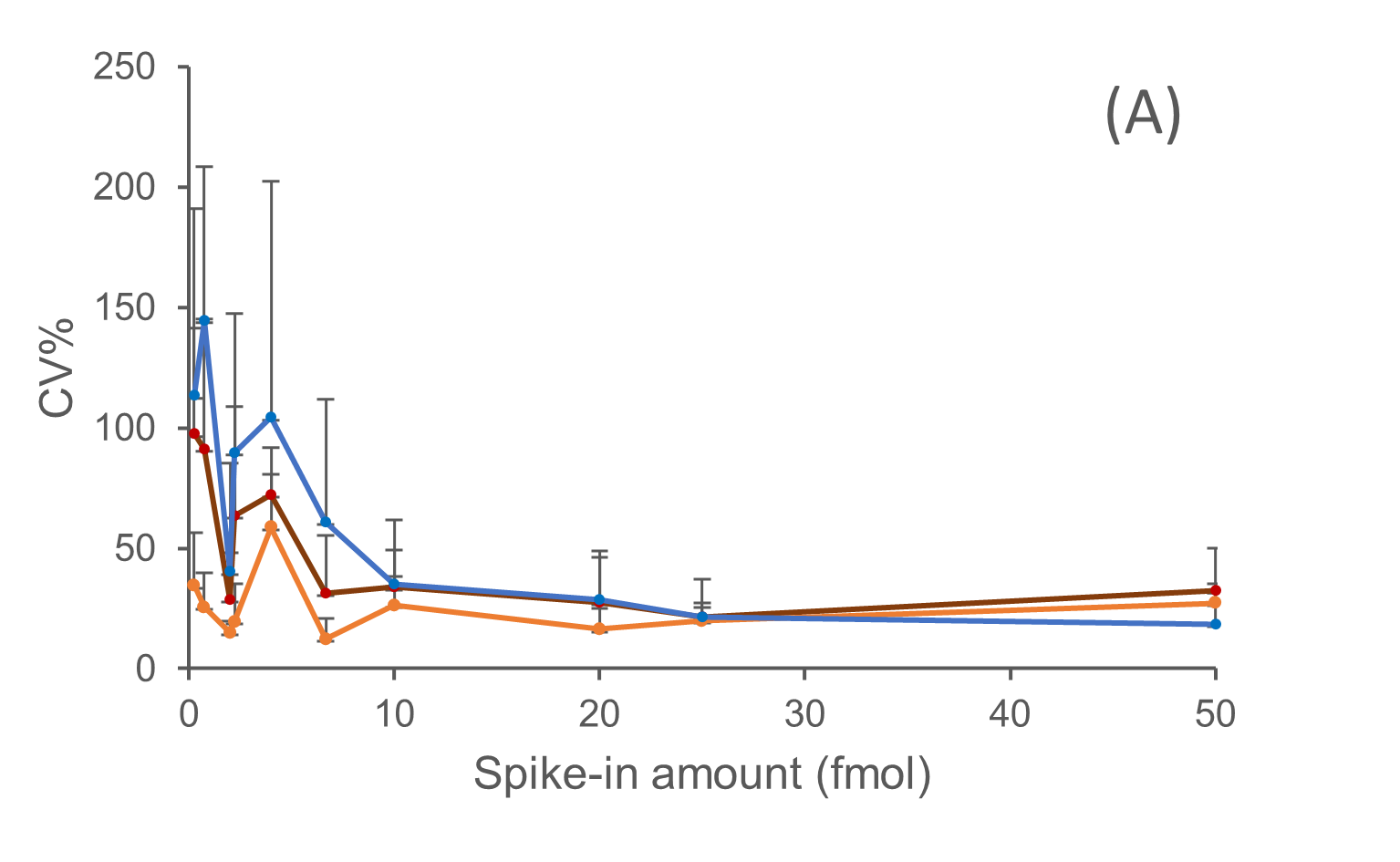


**Fig. S2**. Pairwise comparison-based correlation coefficients between experimental and theoretical UPS ratios across the tested dilutions for (A, B, C) the dataset D2 (Tabb et al. 2010, ref. [28]), and for (D, E, F) dataset D1 (PRIDE repository code PDX002099, ref. [27]), computed based on the LFQ values determined by DDASSQ and Proteome Discoverer^TM^ (PD): (A,D) Box plots and (B, C, D, F) individual measured ratios plotted against the corresponding theoretical values.

**
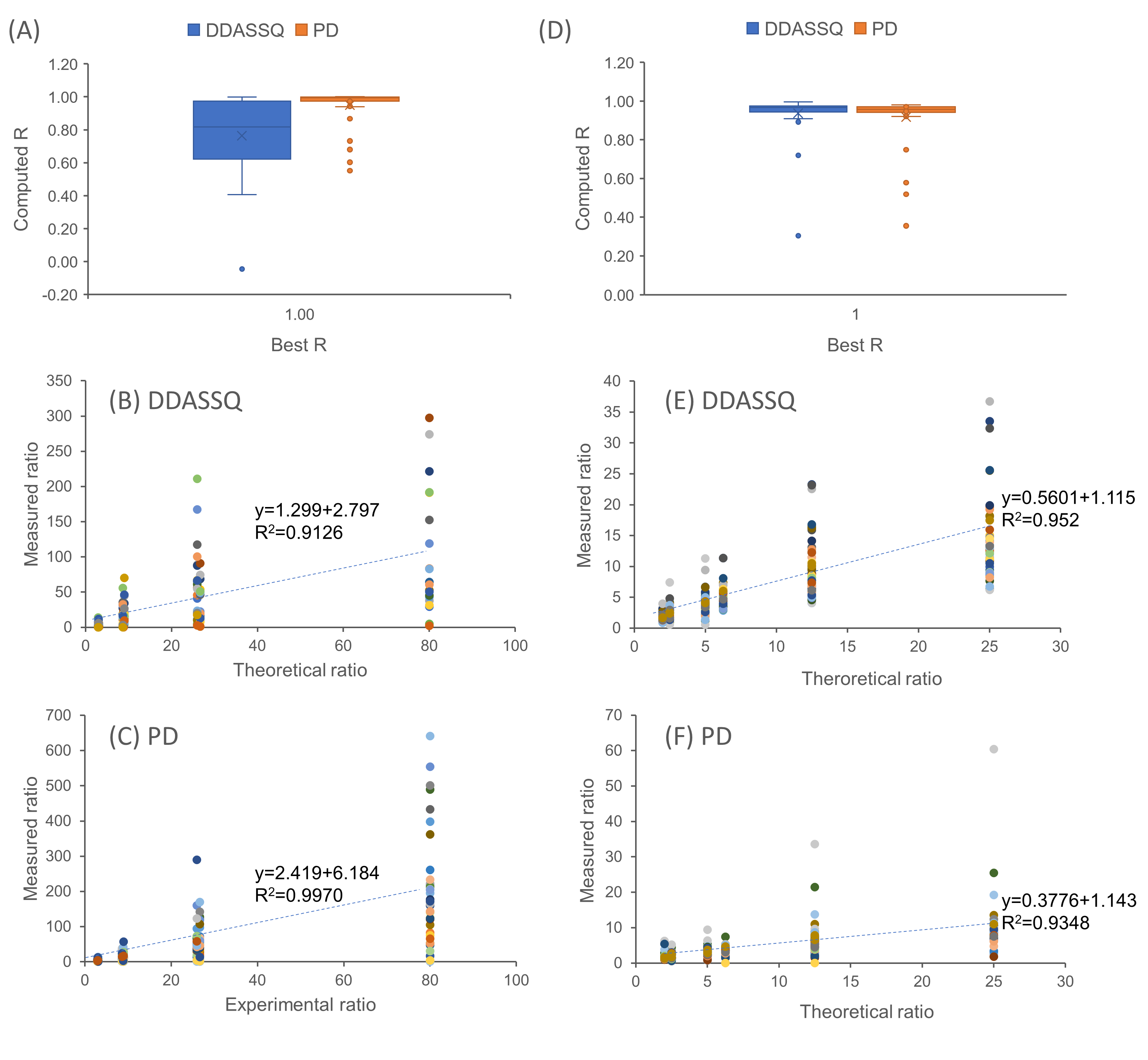
**


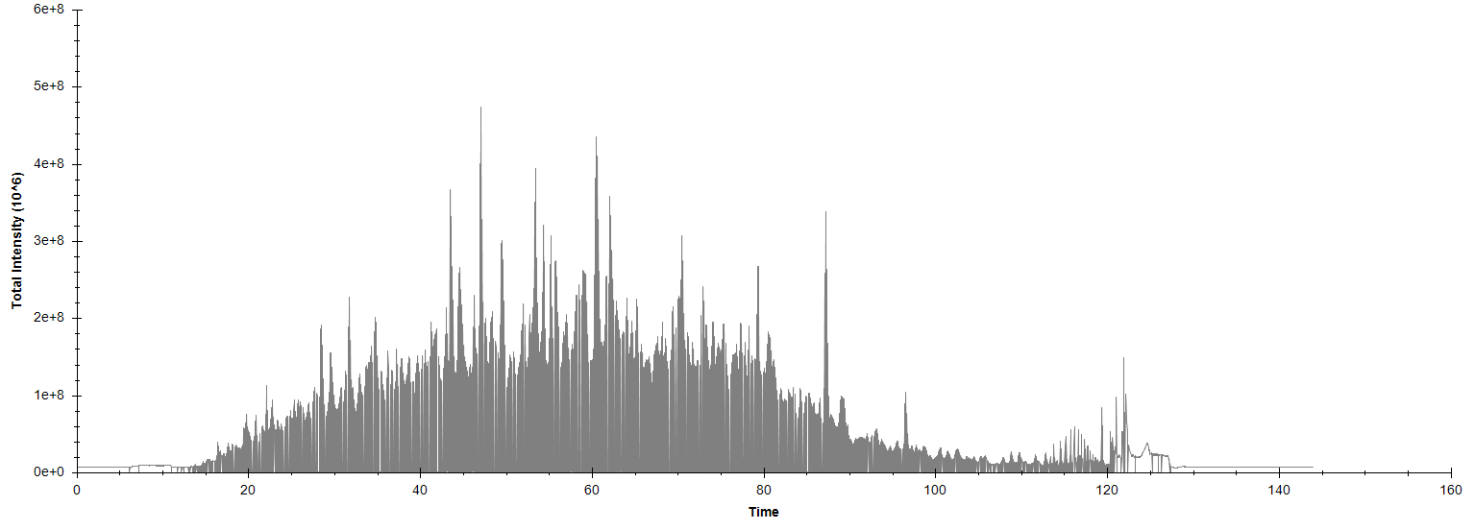
**Fig. S3.** LC-MS chromatographic profile of fraction F1, first replicate


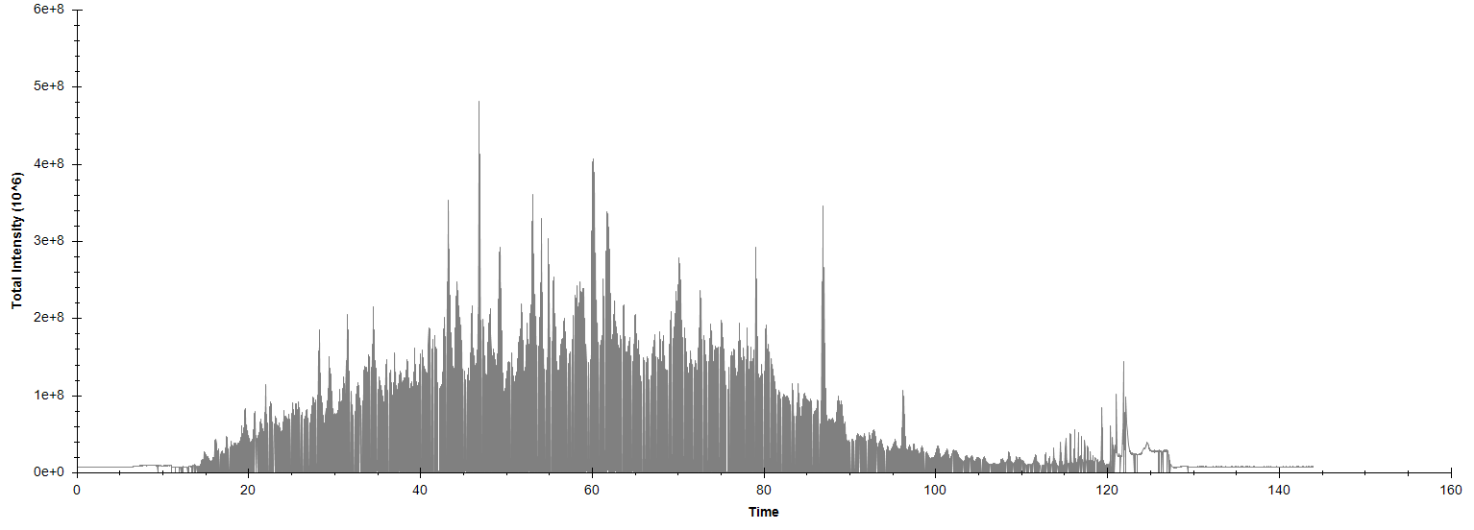
**Fig. S4.** LC-MS chromatographic profile of fraction F1, second replicate

**Fig. S5.** LC-MS chromatographic profile of fraction F2, first replicate

**
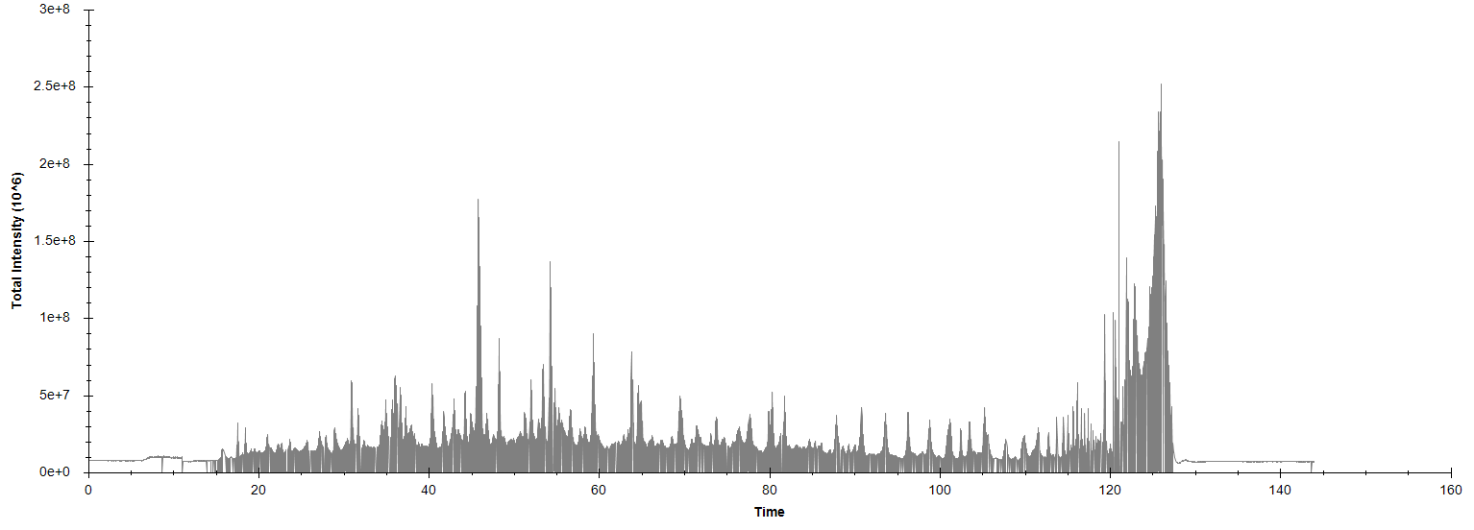
**

**Fig. S6.** LC-MS chromatographic profile of fraction F2, second replicate


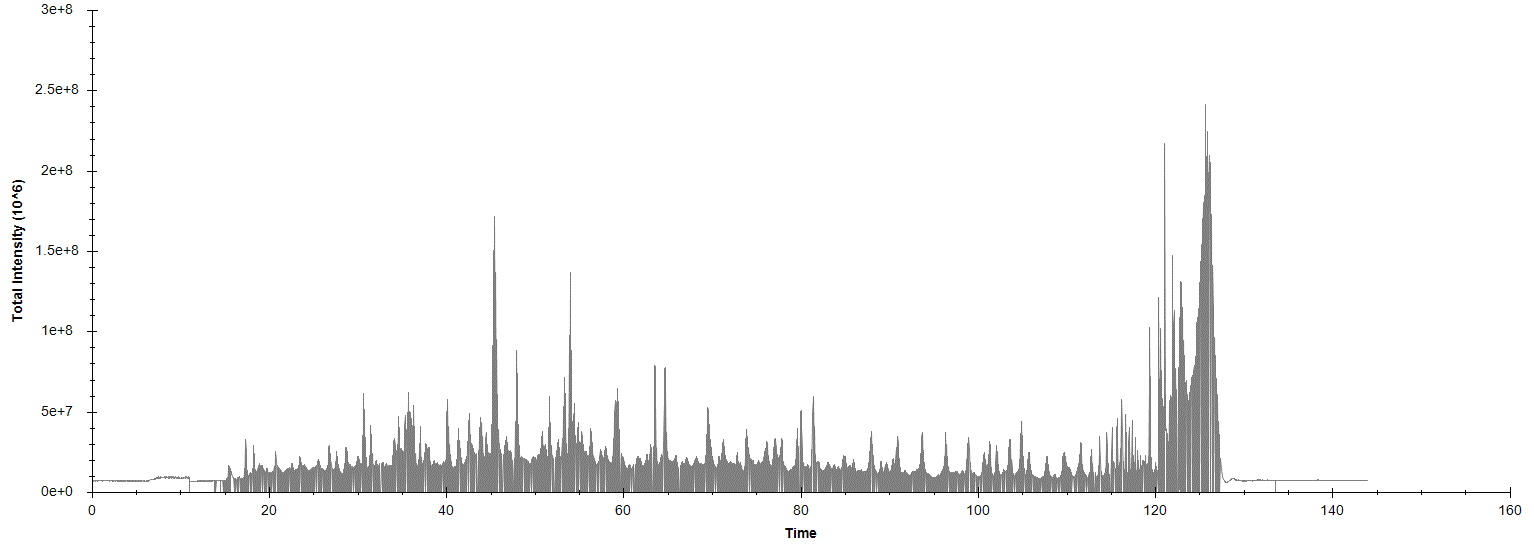


**Appendix 1**

OpenMS workflow nodes parameters. No setting action needed for: ZipLoopStart, ZipLoopEnd, Port to URI, PIA Compiler, URI Port to Variable


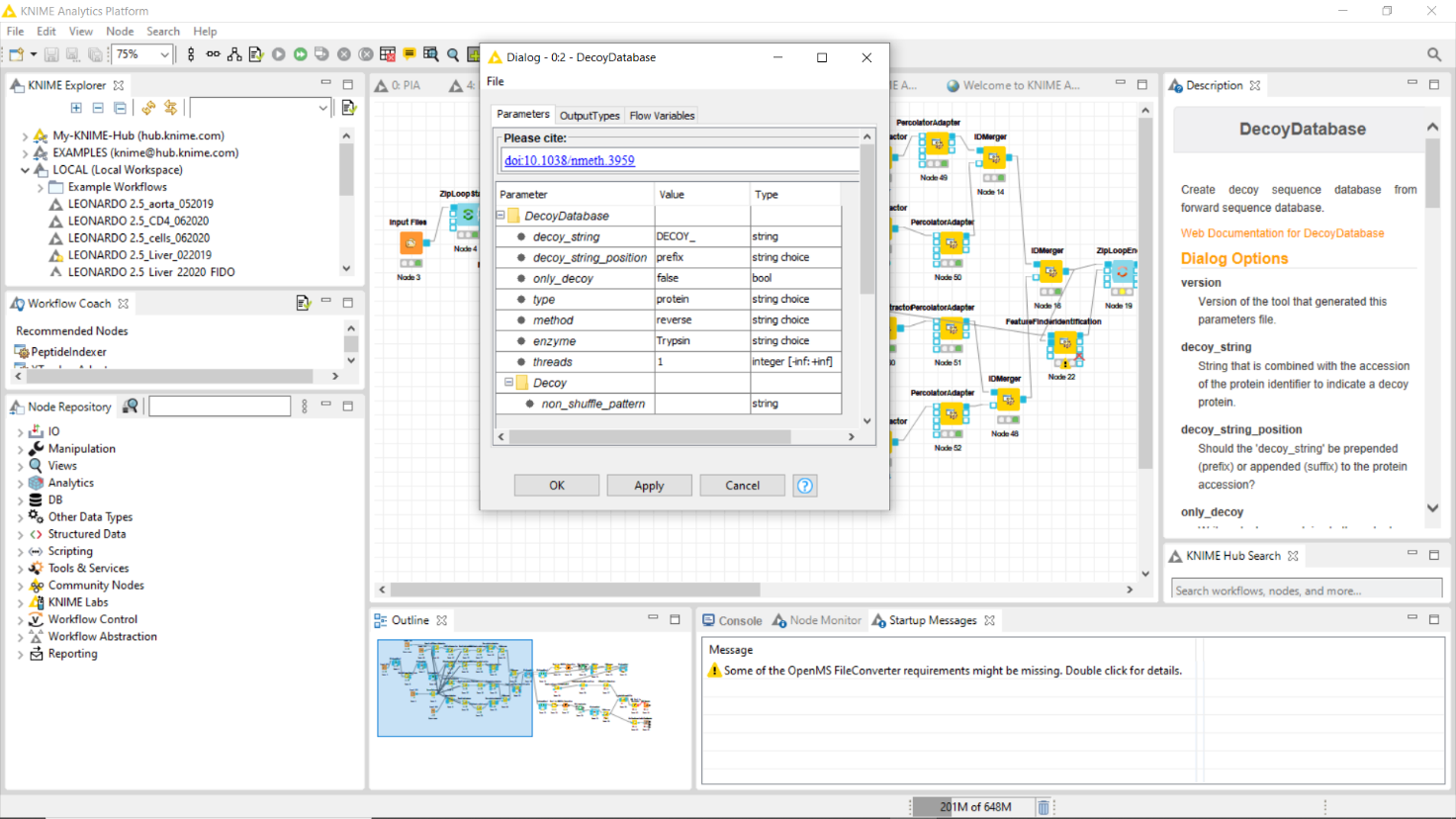

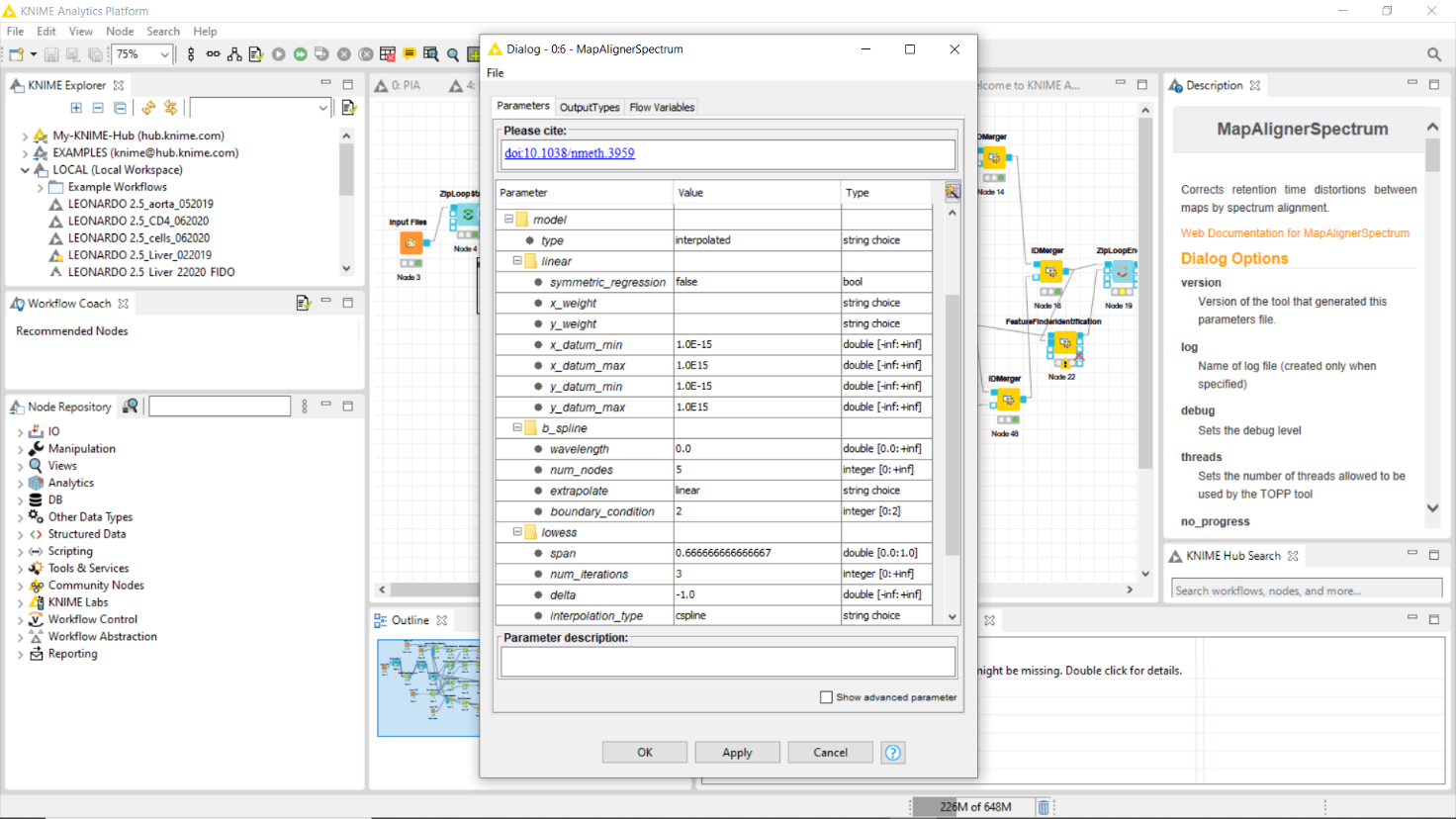
MapAlignerSpectrum DecoyDatabase

SpectraSTSearchAdapter XTandemAdapter


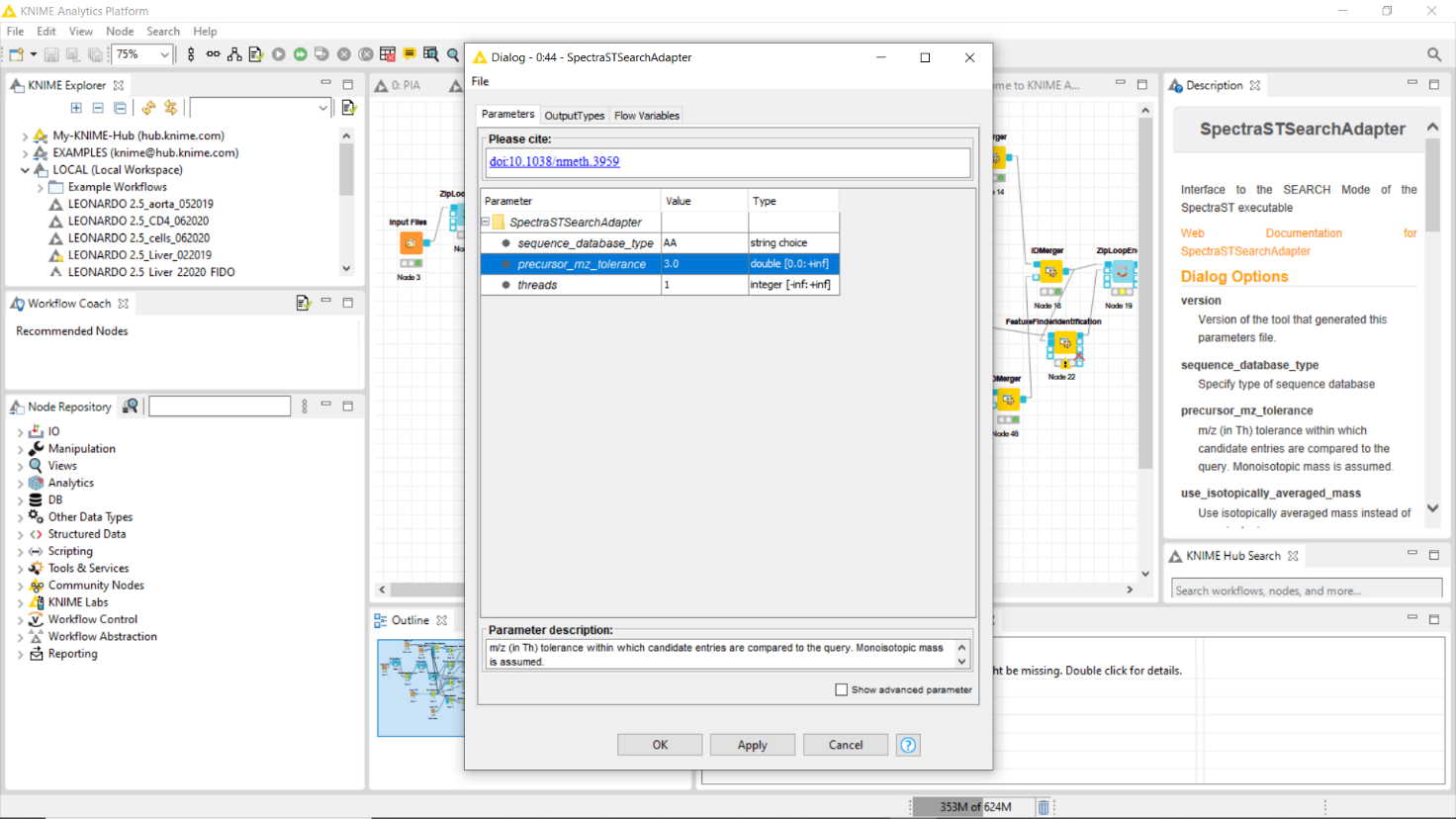

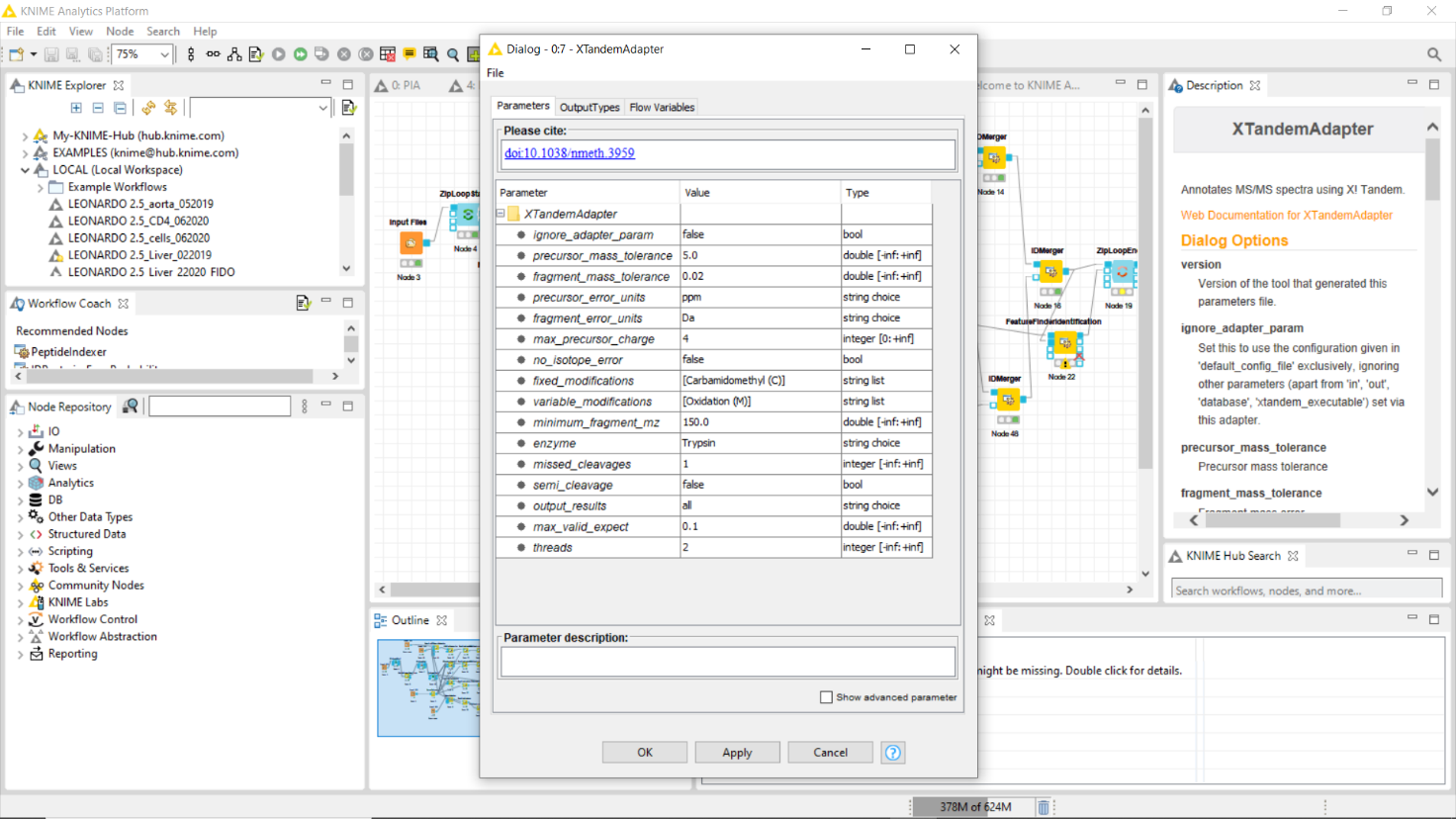


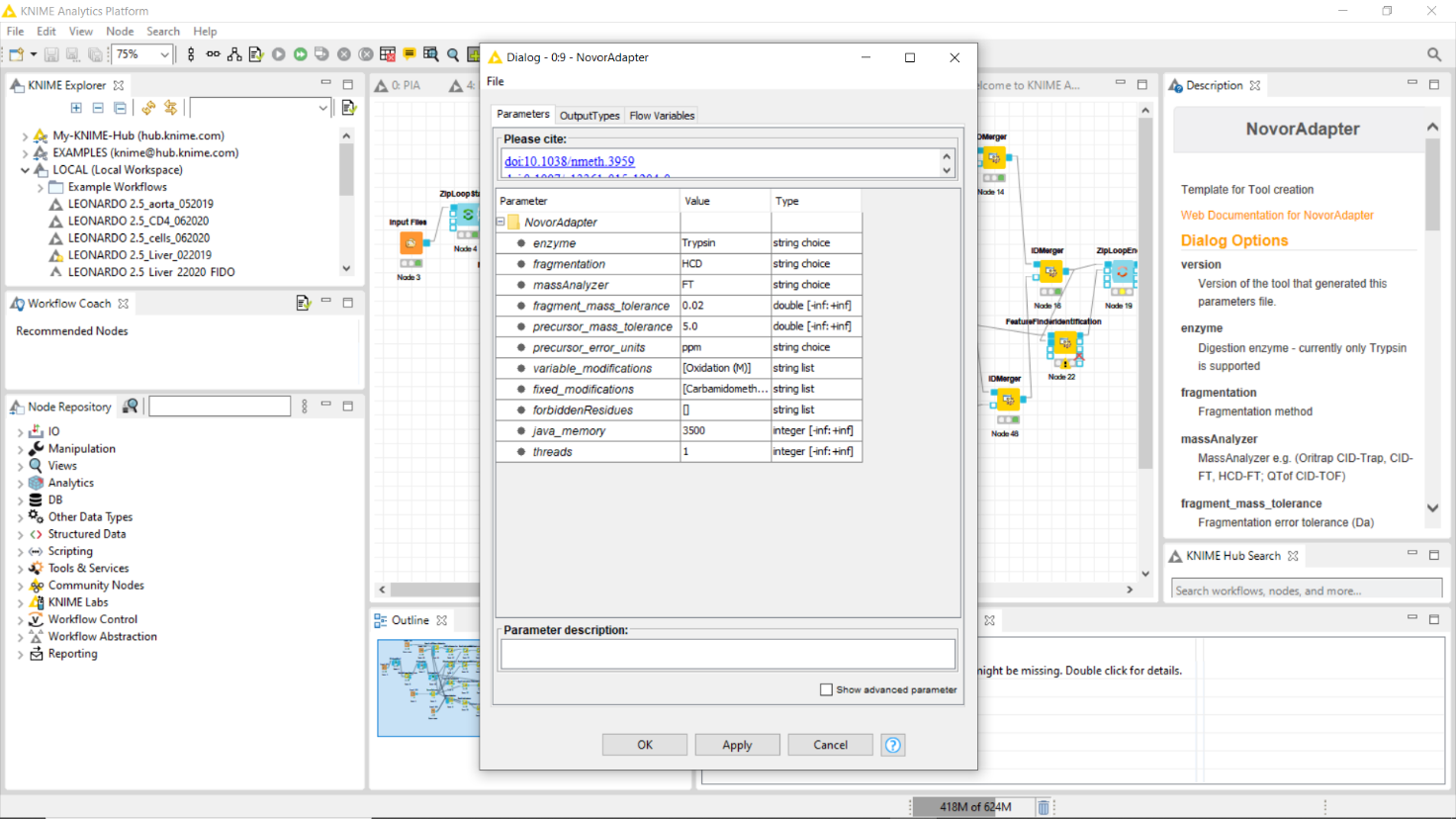

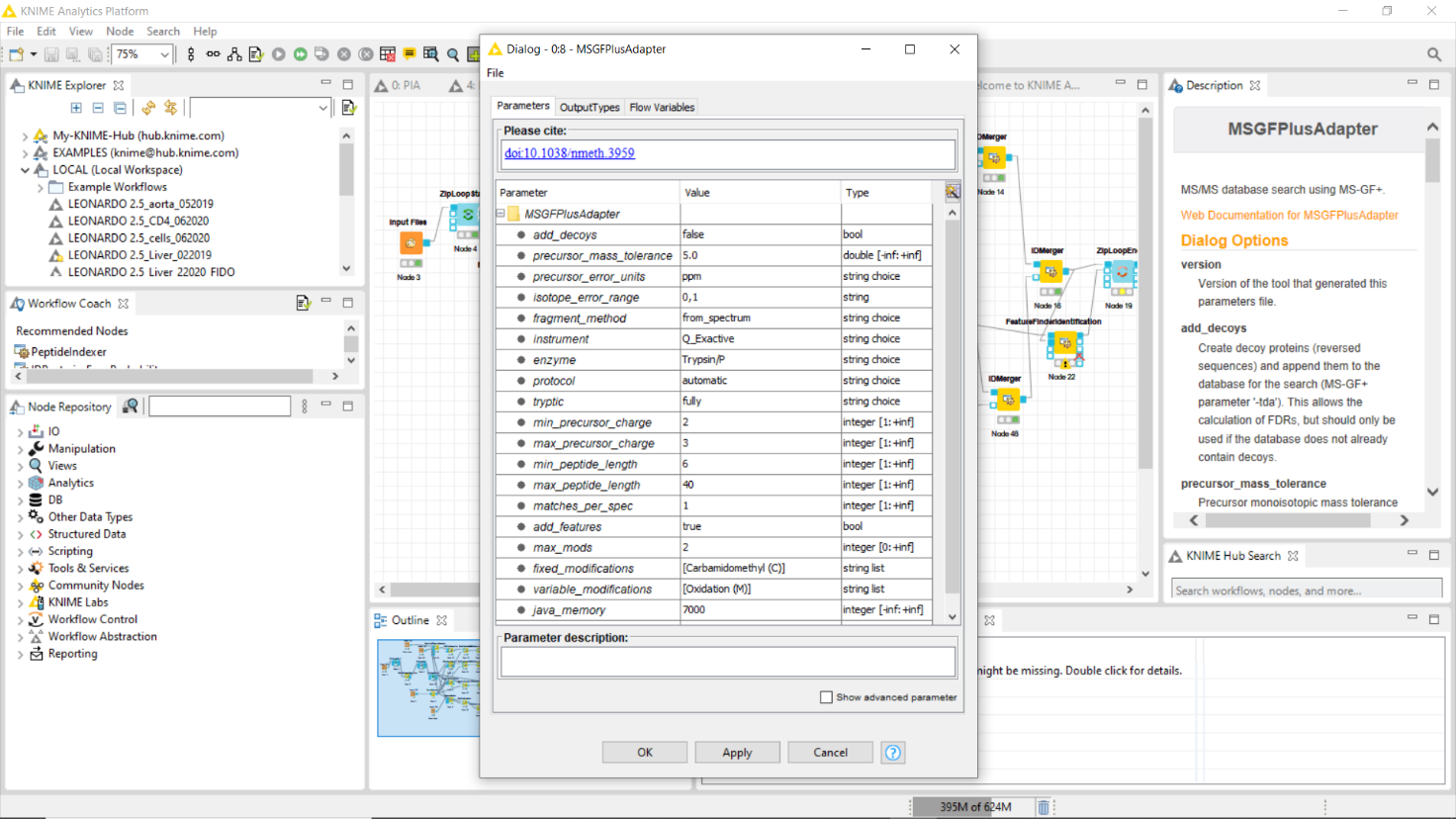
MSGFPlusAdapter NovorAdapter

IDFileMerger


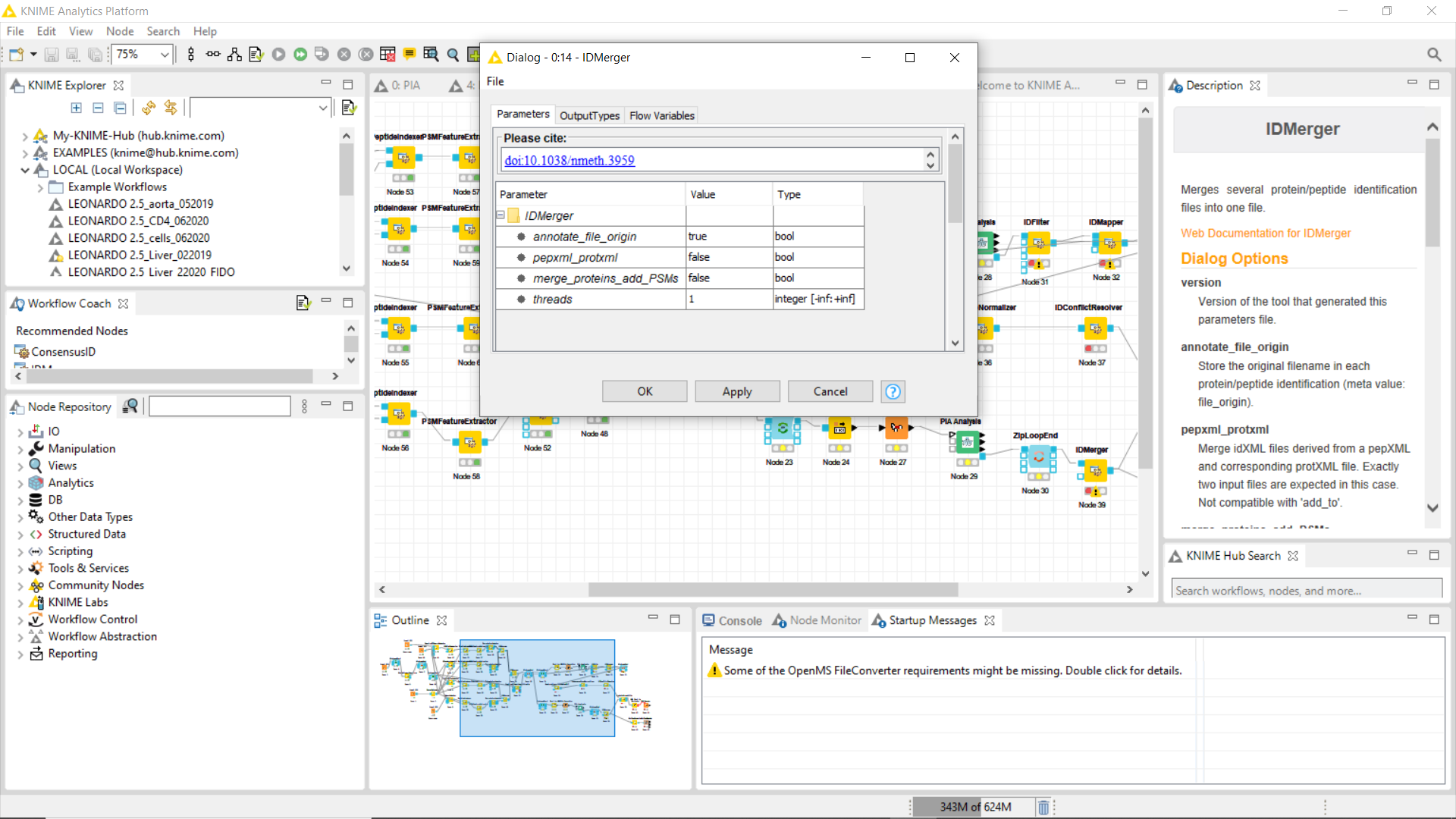


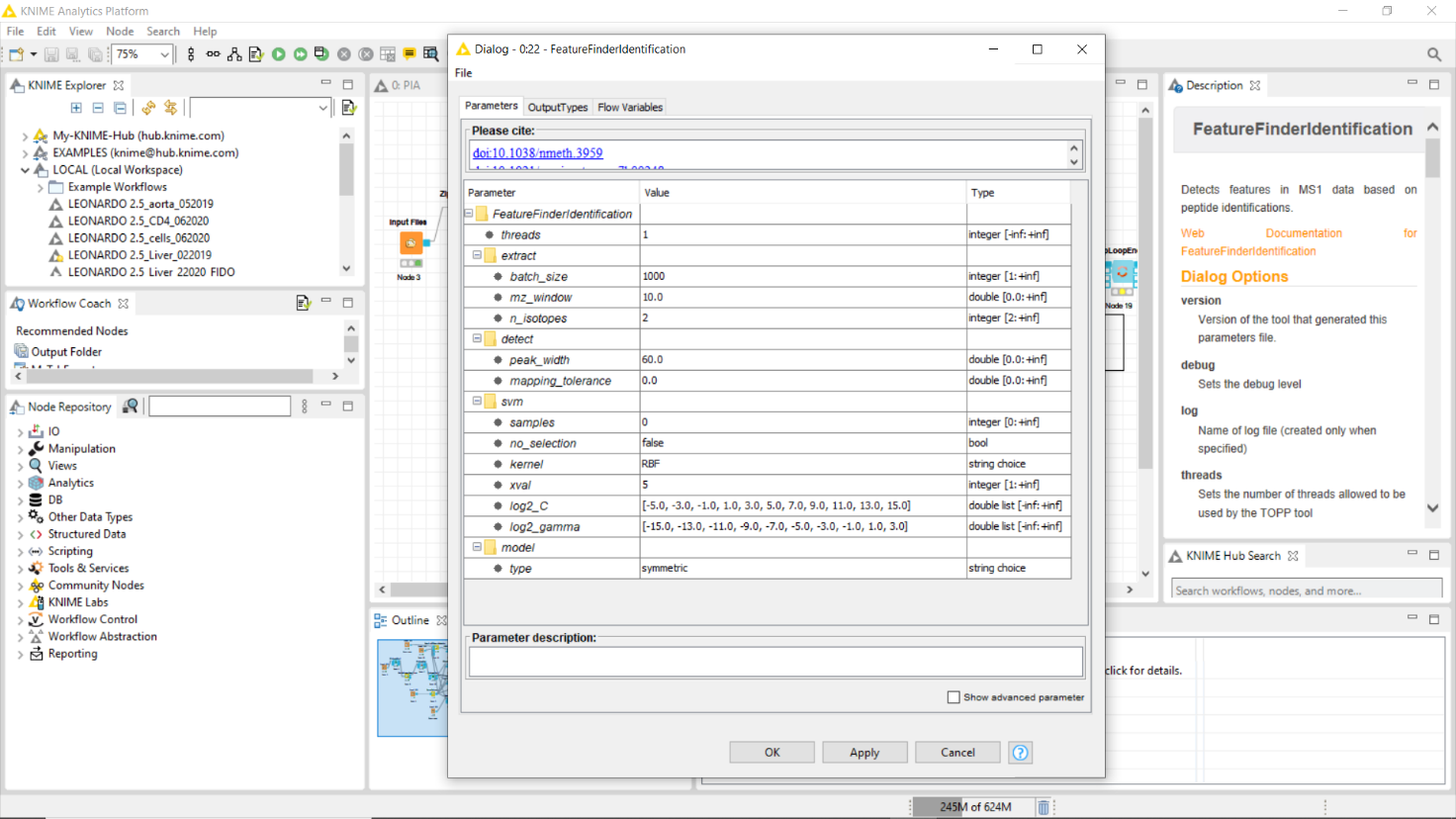
PeptideIndexer FeatureFinderIdentification


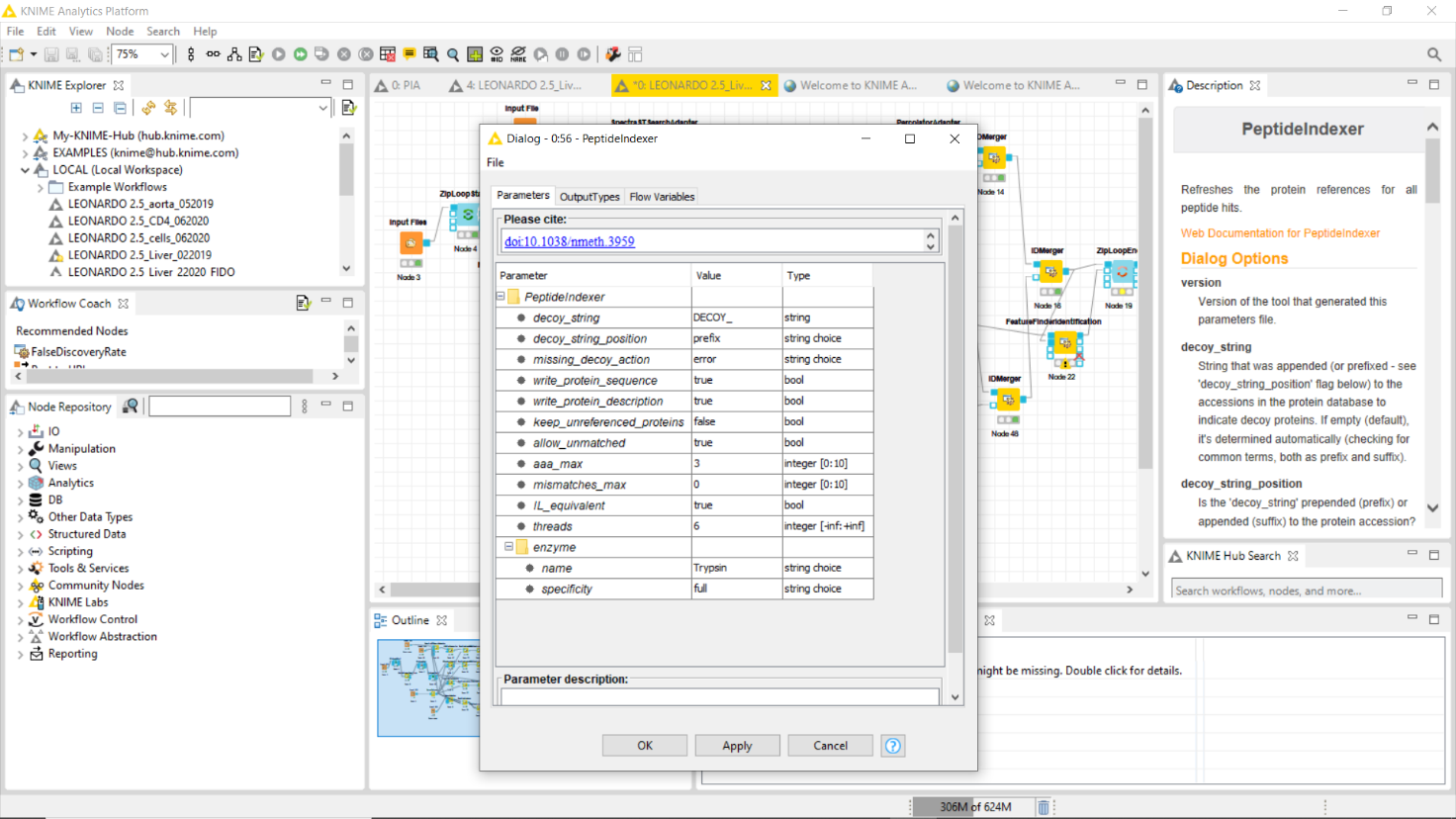


IDFilter


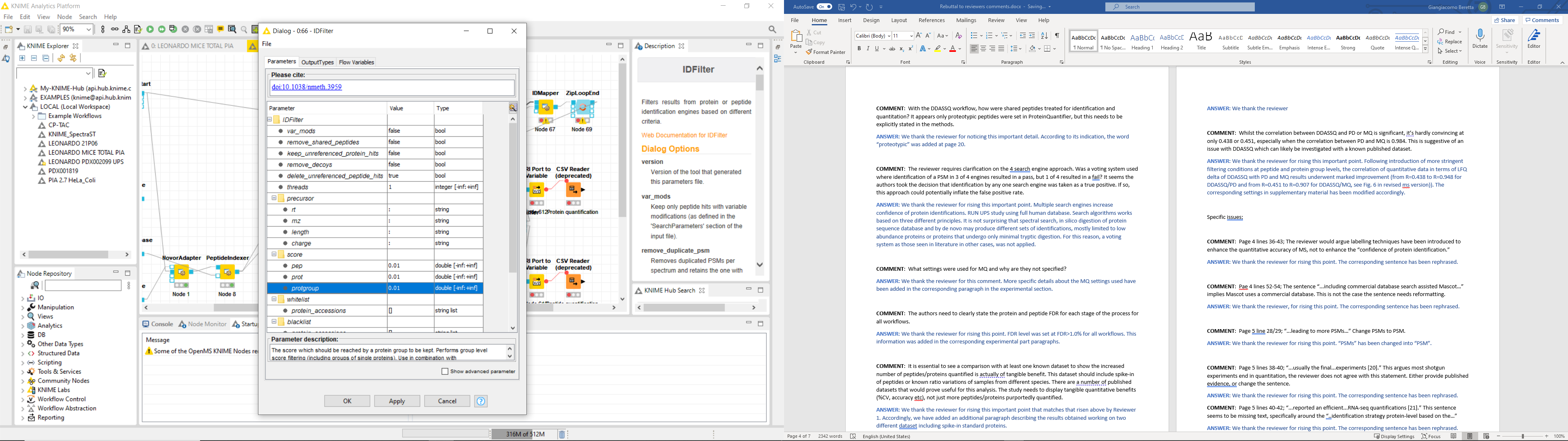


ProteinQuantifier


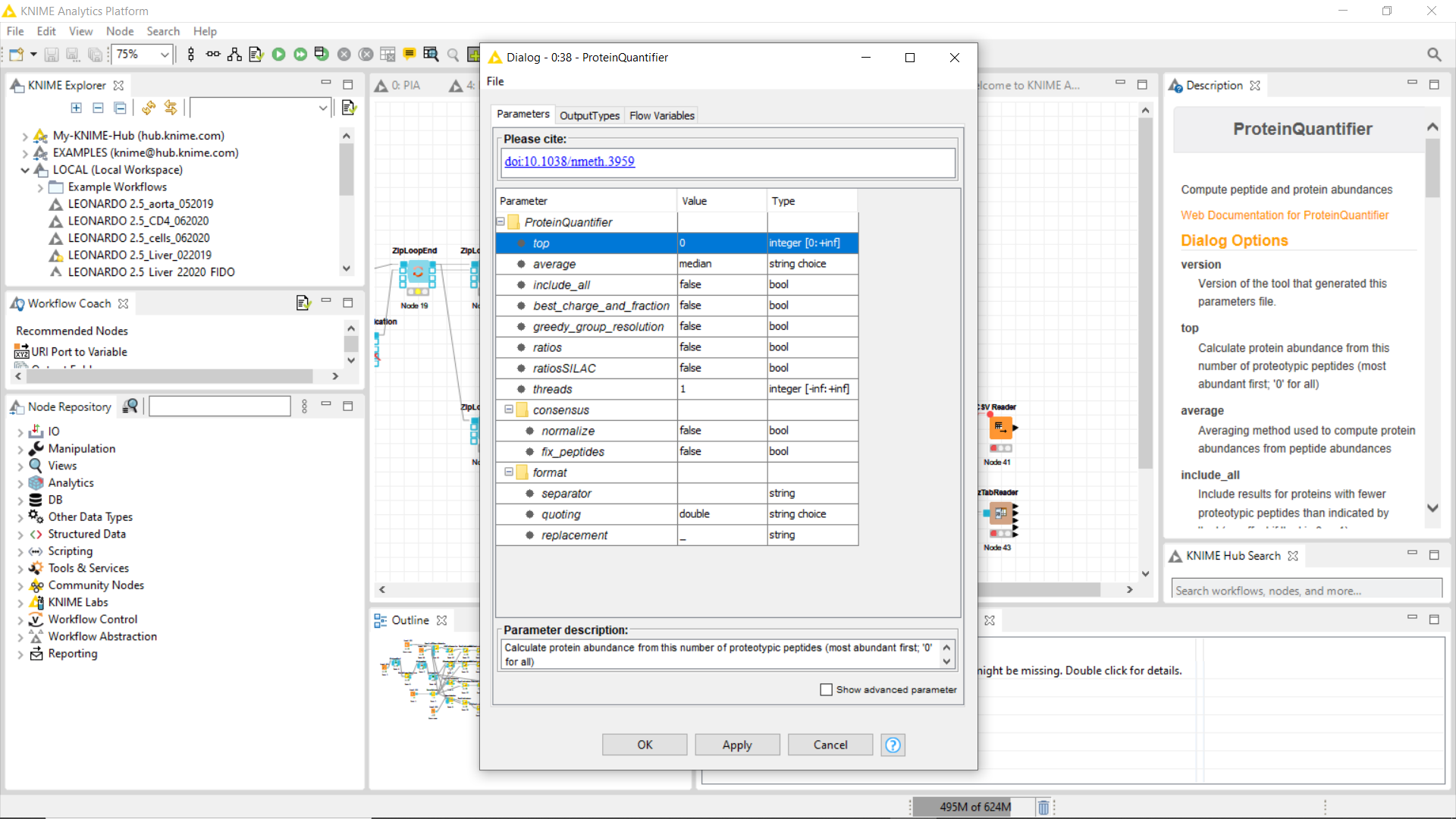


**Appendix 2**

Proteome Discoverer^TM^ data processing workflow.

Processing Step:


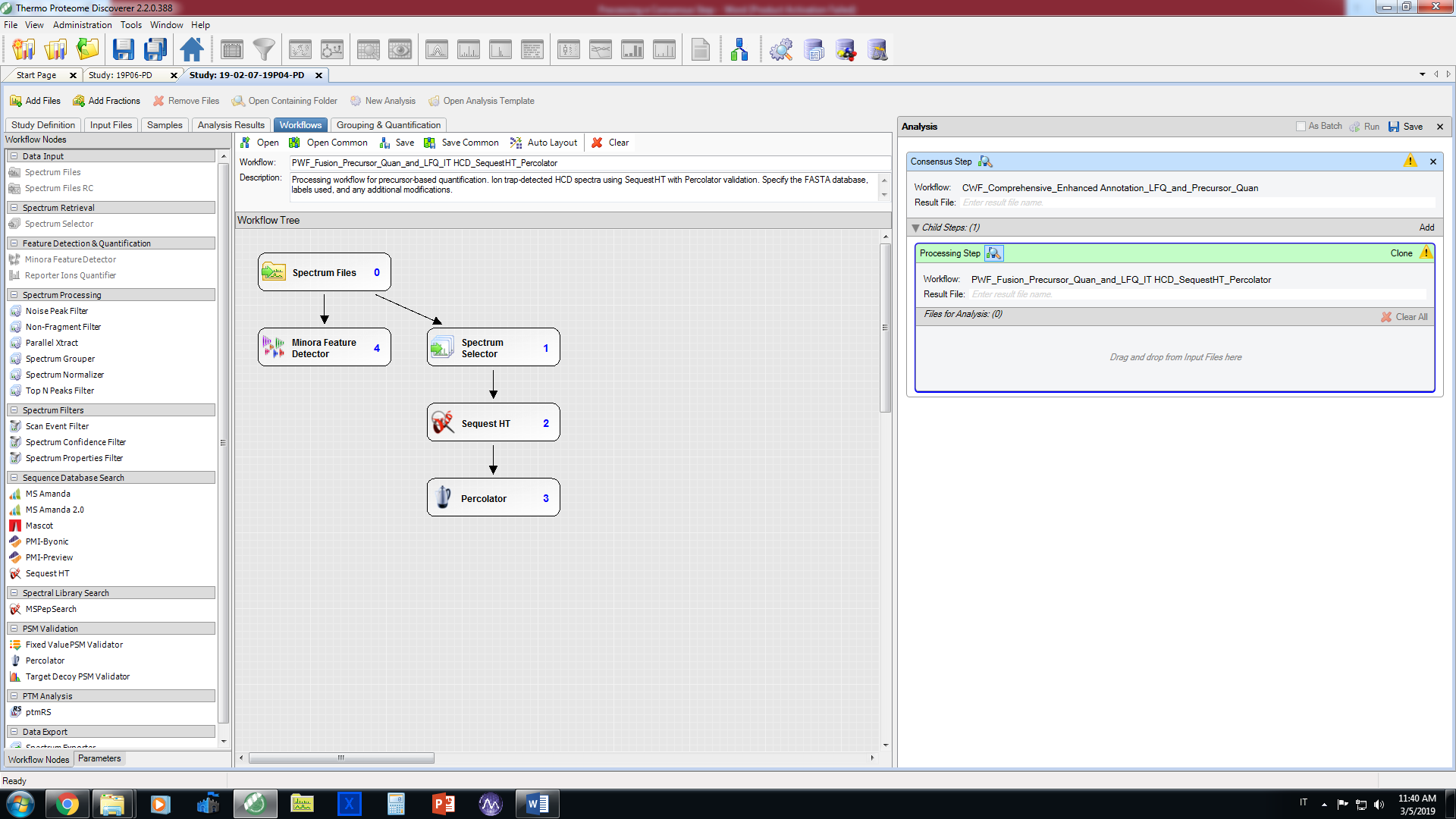


Consensus Step:


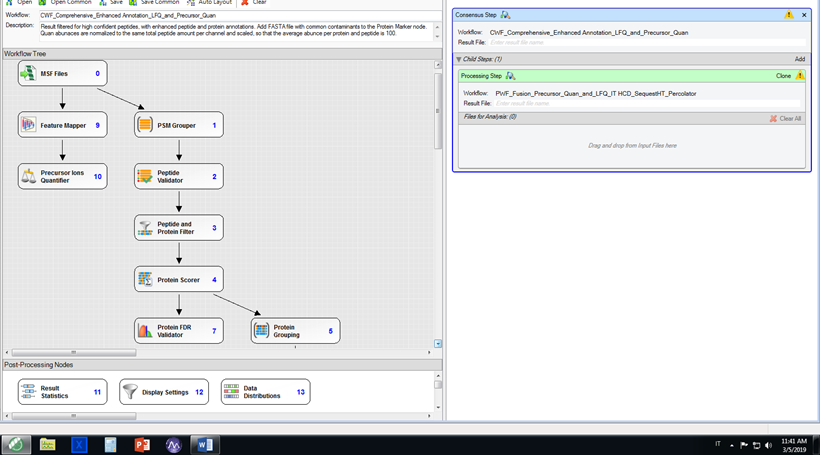

Supplement: Supplementary file 1 — Supporting Information [file PMIC-21-2000319-s001.doc]
